# Bifurcation Analysis on Phase-Amplitude Cross-Frequency Coupling in Neural Networks with Dynamic Synapses

**DOI:** 10.3389/fncom.2017.00018

**Published:** 2017-03-30

**Authors:** Takumi Sase, Yuichi Katori, Motomasa Komuro, Kazuyuki Aihara

**Affiliations:** ^1^Graduate School of Information Science and Technology, The University of TokyoTokyo, Japan; ^2^Institute of Industrial Science, The University of TokyoTokyo, Japan; ^3^The School of Systems Information Science, Future University HakodateHokkaido, Japan; ^4^Center for Fundamental Education, Teikyo University of ScienceYamanashi, Japan

**Keywords:** phase-amplitude cross-frequency coupling, dynamic synapse, excitatory and inhibitory networks, mean field model, bifurcation analysis, MT1SNC bifurcation, discrete-time neuron model

## Abstract

We investigate a discrete-time network model composed of excitatory and inhibitory neurons and dynamic synapses with the aim at revealing dynamical properties behind oscillatory phenomena possibly related to brain functions. We use a stochastic neural network model to derive the corresponding macroscopic mean field dynamics, and subsequently analyze the dynamical properties of the network. In addition to slow and fast oscillations arising from excitatory and inhibitory networks, respectively, we show that the interaction between these two networks generates phase-amplitude cross-frequency coupling (CFC), in which multiple different frequency components coexist and the amplitude of the fast oscillation is modulated by the phase of the slow oscillation. Furthermore, we clarify the detailed properties of the oscillatory phenomena by applying the bifurcation analysis to the mean field model, and accordingly show that the intermittent and the continuous CFCs can be characterized by an aperiodic orbit on a closed curve and one on a torus, respectively. These two CFC modes switch depending on the coupling strength from the excitatory to inhibitory networks, via the saddle-node cycle bifurcation of a one-dimensional torus in map (MT1SNC), and may be associated with the function of multi-item representation. We believe that the present model might have potential for studying possible functional roles of phase-amplitude CFC in the cerebral cortex.

## 1. Introduction

Neurons in the brain, process information through diverse neural dynamics emergent from interactions among neurons via synapses. How neural networks formed by the interactions can generate functional dynamics, such as oscillatory or synchronized activity and more specifically, cross-frequency coupling (CFC), largely remains to be explored.

A variety of oscillatory phenomena in the brain have been studied often through the measurement of the local field potential or an electroencephalogram, both of which include macroscopic information of neural networks (cell assemblies), different from single-unit recordings. The recorded macroscopic neural activity can provide useful indices of distinctive brain functions, where such indices show a possibility that the oscillatory phenomena correlate with the brain functions (Buzsáki and Draguhn, 2004). Further, the recorded neural activity represents various types of oscillatory waveforms and can be categorized according to the frequency, whose bands, for example, theta and gamma bands, can temporally coexist in the same or different brain regions (Steriade, 2001; Csicsvari et al., 2003). Among the sorted frequency bands, neighboring bands recorded in the same brain region may differ with the brain functions (Klimesch, 1999; Kopell et al., 2000; Engel et al., 2001; Csicsvari et al., 2003). The oscillatory frequency is nearly inversely proportional to the power in general as observed from the power spectrum (Freeman et al., 2000). This inversely proportional property of the power suggests that spatially widespread slow oscillations can modulate the local neural activity (Steriade, 2001; Csicsvari et al., 2003; Sirota et al., 2003). Specifically, when the slow and fast oscillatory components interact with each other, CFC phenomena emerge.

The mechanism underlying the oscillatory phenomena is thought to be based on the following three elements: neurons, synapses, and connectivity, all of which are necessary to constitute neural networks and to generate diverse macroscopic oscillations such that sensory input reflecting external environmental input can be correctly encoded in the oscillations.

First, a neuron generates spike trains stochastically and irregularly; that is, the neuron itself may not possess the reliability to generate clear rhythms, although evidence that a single neuron can produce different rhythms has been obtained on the other hand. There exist, at least, two types of neurons in the cerebral cortex, namely, excitatory and inhibitory neurons, which exhibit different response properties (Lux and Pollen, 1966; Connors et al., 1982; Connors and Gutnick, 1990; Kawaguchi and Kubota, 1997; Nowak et al., 2003; Tateno et al., 2004). In particular, an excitatory neuron typically shows a low firing rate whereas an inhibitory neuron shows a high firing rate (Tateno et al., 2004).

The second fundamental element for the oscillatory phenomena is a synapse, intermediating between neurons with its transmission efficacy. It had been considered that the synaptic efficacy is nearly constant over time or changing very slowly; more specifically, peaks of the synaptic current, induced by releases of neurotransmitters from presynaptic vesicles, had been thought to be independent of the timing of neural firings. During the last few decades, many reports have shown, however, that synapses involve fast plasticity mechanisms, enabling neurons with such synapses to generate more flexible dynamics compared to those with ‘static’ synapses. In particular, synapses that exhibit rapid changes in the coupling strength between neurons with a short-term plasticity mechanism are called dynamic synapses (Markram and Tsodyks, 1996; Markram et al., 1998; Thomson, 2000; Wang et al., 2006). Two types of dynamic synapses exist, namely, short-term depression and facilitation synapses, which are characterized by the transiently decreasing ratio of releasable neurotransmitters and by the transiently increasing calcium concentration in presynaptic terminals, respectively. The synaptic transmission efficacy decreases or increases depending on the ratio of the two time constants associated with the recovery from the aforementioned transient decrease or increase (Markram et al., 1998; Thomson, 2000). The distribution of the dynamic synapses in the brain differs among brain regions. For example, many depression synapses appear in the parietal lobe, while many facilitation synapses appear in the prefrontal lobe (Wang et al., 2006).

The third element for generating the oscillatory phenomena is connectivity, which enables neurons to interact with each other and to form a neural network via synapses. Although each neuron fires stochastically and the resulting spike trains do not show clear rhythmicity, the neural network can macroscopically generate rhythmic oscillations. When excitatory neurons aggregate to be structured as a cell assembly receiving input from inhibitory neurons, rhythmic dynamics on networks macroscopically appears and possesses reliability (Yoshimura et al., 2005). In particular, interactions between two types of networks, i.e., excitatory and inhibitory networks, would underlie the emergence of CFC (Aru et al., 2015; Hyafil et al., 2015). Notably, a network composed of the excitatory and inhibitory subnetworks can generate either slow or fast oscillations. Thus, the interaction between this network and another, which is also composed of the excitatory and inhibitory subnetworks, can be one origin of CFC; this coupling structure has been called as bidirectional coupling (Hyafil et al., 2015).

Here, stochastic neural network models have been utilized to elucidate the relationship between irregular spike trains and rhythmic macroscopic oscillations. The process of the macroscopic oscillations generated from such irregular dynamics can be explained by a network receiving the following two types of input: strong external noise and strong recurrent inhibition (Brunel and Hakim, 1999; Brunel and Wang, 2003). The fast repetition of such noisy input causes short-term synaptic depression, and then, the network induces the destabilization of attractors (Cortes et al., 2006) and chaotic oscillations (Marro et al., 2007).

Additionally, the stochastic neural network models with dynamic synapses have been intensively investigated. Synaptic depression triggers a large fluctuation in sustained periods between the up and down states (Mejias et al., 2010), and the level of the synaptic depression changes the property of the sustained activities of these two different states (Benita et al., 2012). Moreover, the synaptic depression contributes to the destabilization of network activity, the generation of an oscillatory state, and the spontaneous state transitions among multiple patterns in an associative memory network (Katori et al., 2013). Additionally, the synaptic depression can be a suitable mechanism to explain critical avalanches in self-organized neural networks (Bonachela et al., 2010). On the other hand, synaptic facilitation enhances the working memory function (Mongillo et al., 2008). Both synaptic depression and facilitation are related to the storage capacity of attractor neural networks (Bibitchkov et al., 2002; Torres et al., 2002; Matsumoto et al., 2007; Mejias and Torres, 2009; Otsubo et al., 2011; Mejias et al., 2012). Bibitchkov et al. have shown that depression synapses may reduce the storage capacity (Bibitchkov et al., 2002). Torres et al. investigated this negative effect of depression synapses on the storage capacity in general (Torres et al., 2002). However, Matsumoto et al. argued that the storage capacity is not influenced by synaptic depression, when noise is not considered in the network (Matsumoto et al., 2007). Otsubo et al. reported that a network with both depression synapses and the noise would reduce the storage capacity (Otsubo et al., 2011). Then, Mejias and Torres found that the combination of depression and facilitation synapses can enhance the storage capacity (Mejias and Torres, 2009) and furthermore, Mejias et al. generated phase diagrams, indicating that synaptic facilitation enlarges the memory phase region (Mejias et al., 2012). Further, dynamic synapses play a role in stochastic resonance, where a weak input signal to a network can be detected in an output signal under certain conditions (Pantic et al., 2003; Mejias and Torres, 2008, 2011; Torres et al., 2011; Pinamonti et al., 2012; Torres and Marro, 2015). Pantic et al. have shown that a neuron with depression synapses is capable of detecting noisy input signals with a wider frequency range, compared to one with static synapses, under a certain firing threshold (Pantic et al., 2003). Mejias and Torres found that the inclusion of facilitation dynamics in depression synapses would enhance the detection performance (Mejias and Torres, 2008). Moreover, they have shown that such combination of depression and facilitation synapses can generate two suitable noise levels to detect input signals; where it has been argued that this bimodal resonance is caused by the interplay between the adaptively varying firing threshold and the dynamic synapses (Mejias and Torres, 2011). Torres et al. demonstrated that a model with this interplay can predict experimental data of stochastic resonance (Torres et al., 2011). Furthermore, Pinamonti et al. demonstrated that stochastic resonance is well enhanced near phase transitions among patterns in an associative memory network (Pinamonti et al., 2012). Then, Torres and Marro generated a detailed phase diagram embedding many patterns associated with stochastic resonance, such that multiple noise levels are well responsible for optimizing input signals (Torres and Marro, 2015). Additionally, it has been reported that the combination of depression and facilitation synapses contributes to flexible information representation, from the viewpoint of both data analysis and mathematical modeling (Katori et al., 2011).

In particular, the stochastic neural network models and the corresponding mean field models have been effectively used for analyzing the properties of neural networks, including those with dynamic synapses (Pantic et al., 2002; Torres et al., 2007; Igarashi et al., 2010; Katori et al., 2012). While Pantic et al. and Torres et al. have derived mean field models by taking the population average, with respect to stochastic variables, based on the assumption of ergodicity (Pantic et al., 2002; Torres et al., 2007), Igarashi et al. and Katori et al. have recently introduced dynamic mean field theory in which two types of mean field models, i.e., microscopic and macroscopic mean field models, are in turn derived (Igarashi et al., 2010; Katori et al., 2012).

However, one of the key oscillatory phenomena, CFC described above, has not been well understood, although this coupling phenomenon could contribute to complex information processing in the brain, thanks to the presence of oscillations with two different timescales. On the other hand, much attention to phase-amplitude CFC has been attracted, because it has been suggested that this kind of CFC plays a crucial role in adjusting neural communications among distant brain regions (Canolty et al., 2006; Jansen and Colgin, 2007; Canolty and Knight, 2010). To elucidate possible mechanisms generating the CFC, the continuous-time neuron models have been investigated so far (Malerba and Kopell, 2012; Fontolan et al., 2013), but little is known about discrete-time neuron models, which have been shown to be suitable for simulating, reproducing, and predicting neural phenomena in the brain (Rulkov, 2002; Ibarz et al., 2011). Thus, in this study, we extend the previously proposed discrete-time network model (Katori et al., 2012), which only includes excitatory neurons, to that including also inhibitory neurons, and clarify the bifurcation structure underlying the phase-amplitude CFC.

**Figure 1:**
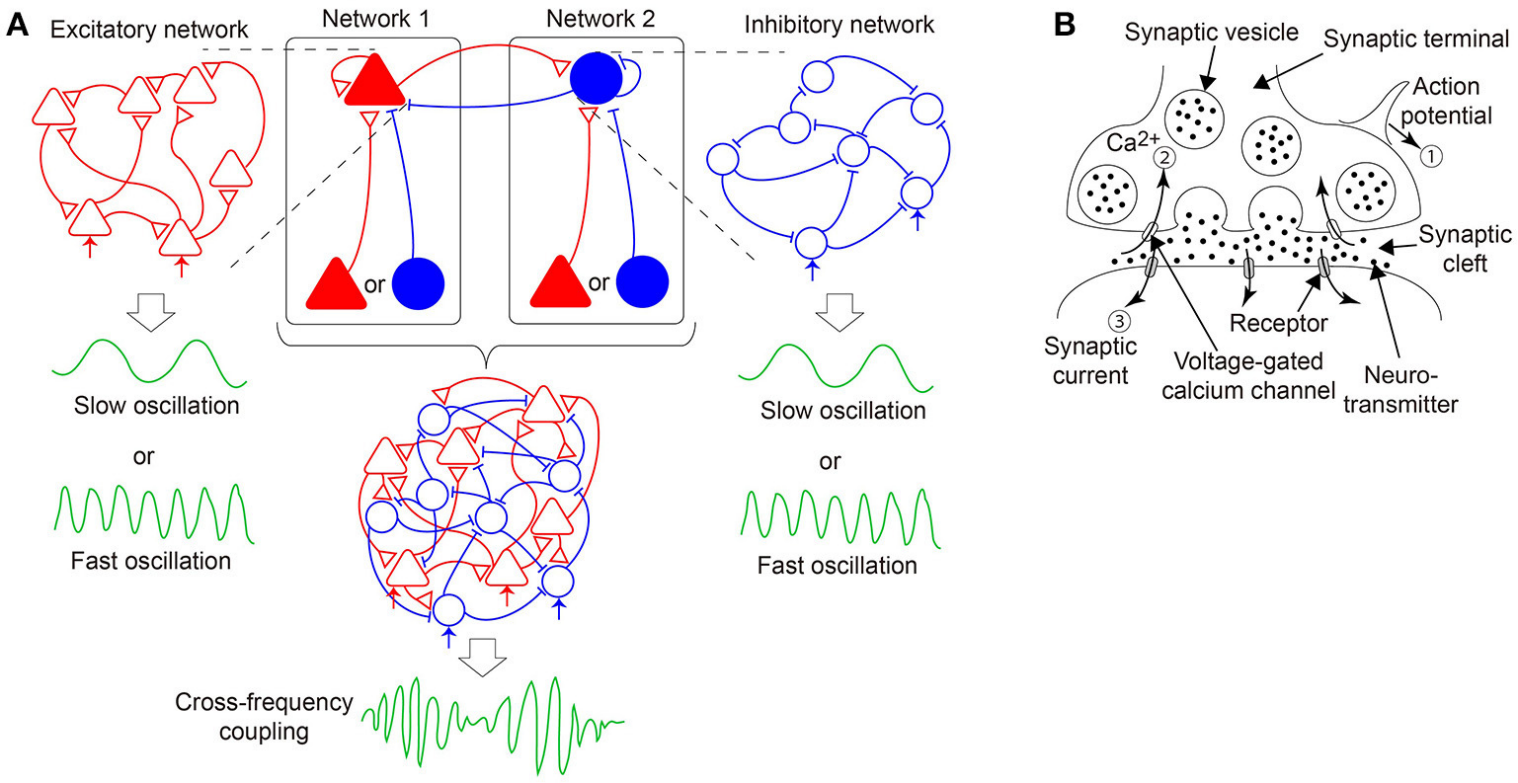
**A hypothesis of emergent dynamics considered in the present model**. **(A)** The cross-frequency coupling (CFC) phenomena can emerge by the bidirectional coupling between networks 1 and 2, both of which consist of excitatory and inhibitory neurons interacting via dynamic synapses and generate either slow or fast oscillations. In network 1 (2), an excitatory (inhibitory) population, indicated by the red filled triangles (the blue filled circles), receives its recurrent connection and input from either another excitatory or inhibitory population. Network 1 (2) is equivalent to a pure excitatory (inhibitory) network since the input can be regarded as external input, depicted in the red (blue) arrows. **(B)** A mechanism of synaptic transmission with short-term plasticity. The action potential (①) of the presynaptic neuron causes a transient decrease in the releasable neurotransmitters and a transient increase in the calcium concentration (②) and triggers the synaptic current (③) on the postsynaptic membrane. If many action potentials arrive successively, and time constants of the transient decrease and increase differ considerably, the synaptic current reflects the property of the dynamic synapses, namely, short-term synaptic depression or facilitation.

In this study, we hypothesize that the CFC is generated by the bidirectional coupling between the two subnetworks as described above, where one subnetwork includes an excitatory population while the other includes an inhibitory population, both of which receive input from another excitatory or inhibitory population and generate slow or fast oscillations (Figure 1A) (White et al., 2000; Kramer et al., 2008; Roopun et al., 2008; Hyafil et al., 2015). Each subnetwork can be regarded as a pure excitatory or a pure inhibitory network because the unidirectional input may be equivalent to change of the neural firing threshold. Therefore, hereinafter, we call the above two subnetworks as an excitatory network/subnetwork and an inhibitory network/subnetwork, respectively, for the sake of simplicity (Figure 1A). Accordingly, we focus on a stochastic network composed of excitatory and inhibitory neurons with dynamic synapses. Furthermore, the proposed model considers the decay process of the synaptic current. We analyze a macroscopic mean field model reproducing the overall network dynamics associated with the stochastic model. Upon the adjustment of parameters specifying the properties of the synaptic current and dynamic synapses, rich bifurcation structures of the network dynamics are expected to be found. In the following sections, first, we describe a network model with stochastic neurons connected via dynamic synapses. Then, we derive a macroscopic mean field model capturing the macroscopic dynamics of the network model. Subsequently, we analyze the bifurcation structures of the present model and illustrate various solutions included in the dynamical systems of not only an excitatory or an inhibitory network, but also a network composed of both the excitatory and the inhibitory subnetworks. Finally, we discuss the dynamical properties revealed from the standpoint of neuroscience, and consider possible future directions of this research.

## 2. Materials and methods

This section consists of three parts. The first part describes the mechanism of signal transmission on synapses with short-term plasticity. The second part explains the stochastic model, composed of excitatory and inhibitory binary neurons and dynamic synapses. The third part introduces the mean field theory in which the stochastic model is converted into the corresponding microscopic and macroscopic mean field models. The microscopic mean field approximation is used for extracting the average neural activity on realization of stochastic variables. In contrast, the macroscopic mean field approximation is used for extracting the average neural activity over population dynamics, and thereby, a low-dimensional discrete-time dynamical system is derived, such that we can identify bifurcation structures.

### 2.1. Mechanism of synaptic transmission

Here, we describe the signal transmission mechanism on dynamic synapses with the short-term plasticity. This mechanism can be explained by the following three processes (Figure 1B): First, an action potential is generated on the presynaptic neuron and transmitted to the terminals of the synapses (①). Second, voltage-dependent calcium channels are opened by the action potential, and calcium ions flow into the synaptic terminals via the channels (②). Third, chemical reactions with these calcium ions cause the fusion of the presynaptic membrane and synaptic vesicles, including neurotransmitters, which are released into the synaptic cleft. The released neurotransmitters attach to the postsynaptic membrane, and then, a synaptic current is generated (③). The amplitude of the synaptic current decays with a certain time constant, dependent on the properties of the postsynaptic receptors.

Arrival of many action potentials within a short period of time causes a transient decrease or increase in the efficacy of the synaptic transmission. This is because of changes in the amount of neurotransmitters and in the calcium concentration in the presynaptic terminal. Finally, the synaptic vesicles are retrieved and the neurotransmitters are restored in the reusable synaptic vesicles (Markram and Tsodyks, 1996; Markram et al., 1998; Thomson, 2000; Wang et al., 2006).

### 2.2. Model

We consider a discrete-time neural network model composed of the excitatory (E) and the inhibitory (I) subnetworks, which consist of *N*_E_ excitatory and *N*_I_ inhibitory neurons, respectively. The state of the *i*th neuron belonging to the network ξ (ξ∈{E, I}) at time *t*, denoted by $s_{i}^{\xi }\left(t\right)$, indicates either a quiescent state $\left[s_{i}^{\xi }\left(t\right)=0\right]$ or an active state $\left[s_{i}^{\xi }\left(t\right)=1\right]$. The state of the neuron is stochastically determined by total input for the neuron. This stochasticity is reflecting the biologically observed noisy neural activity. The evolution of the neuronal state is described as follows:

(1) $$\begin{array}{l} \text{Prob}\left[s_{i}^{\xi }\left(t+1\right)=1\right]=g_{\beta^{\xi }}\left[h_{i}^{\xi }\left(t\right)\right], \end{array}$$

(2) $$\begin{array}{l} g_{\beta^{\xi }}\left[h_{i}^{\xi }\left(t\right)\right]=\frac{1}{2}\left\{1+\tanh\left[\beta^{\xi }h_{i}^{\xi }\left(t\right)\right]\right\}, \end{array}$$

where $h_{i}^{\xi }\left(t\right)=\overset{N_{\xi }}{\underset{j\neq i}{\sum }}\left[J_{ij}^{\xi \xi }a_{j}^{\xi }\left(t\right)\right]+\overset{N_{\eta }}{\underset{j=1}{\sum }}\left[J_{ij}^{\xi \eta }a_{j}^{\eta }\left(t\right)\right]+I^{\xi }$ with η∈{E, I|η≠ξ}. The variables $h_{i}^{\xi }\left(t\right)$ and $a_{j}^{\xi }\left(t\right)$ represent the total input for the *i*th neuron on network ξ and the synaptic activity with short-term plasticity, respectively; 1/β^ξ^ = *T*^ξ^ denotes the noise intensity; $J_{ij}^{\xi \xi }$, $J_{ij}^{\xi \eta }$, and *I*^ξ^ indicate the weight of the recurrent coupling from the *j*th neuron to the *i*th neuron on network ξ, the weight of the unidirectional coupling from the *j*th neuron on network η to the *i*th neuron on network ξ, and the external input, respectively.

The signal transmission mechanism in synapses with the short-term plasticity can be modeled by the synaptic activity $a_{i}^{\xi }\left(t\right)$, and two variables, $x_{i}^{\xi }\left(t\right)$ and $u_{i}^{\xi }\left(t\right)$, denoting the ratio of releasable neurotransmitters and the calcium concentration, respectively. The synaptic activity, $a_{i}^{\xi }\left(t\right)$, increases with the presynaptic neural activity. This increase is proportional to the product of $x_{i}^{\xi }\left(t\right)$ and $u_{i}^{\xi }\left(t\right)$, which represents the synaptic transmission efficacy (Markram et al., 1998; Mongillo et al., 2008; Mejias and Torres, 2009). If there is no synaptic activation, $a_{i}^{\xi }\left(t\right)$ converges to its steady state $a_{i}^{\xi }\left(t\right)=0$ with the time constant $\tau_{\text{a}}^{\xi }$. The ratio $x_{i}^{\xi }\left(t\right)$ of releasable neurotransmitters decreases with the presynaptic activation; this decrease is proportional to $u_{i}^{\xi }\left(t\right)$. Then, $x_{i}^{\xi }\left(t\right)$ returns to its steady state $x_{i}^{\xi }\left(t\right)=1$ with the time constant $\tau_{\text{R}}^{\xi }$. The variable $u_{i}^{\xi }\left(t\right)$ increases when a presynaptic neuron is activated, and returns to its steady state $u_{i}^{\xi }\left(t\right)=U_{\text{se}}^{\xi }$ with the time constant $\tau_{\text{F}}^{\xi }$; this increase is proportional to $U_{\text{se}}^{\xi }$. The dynamics described above is summarized as follows:

(3) $$\begin{array}{l} a_{i}^{\xi }\left(t+1\right)=a_{i}^{\xi }\left(t\right)-\frac{a_{i}^{\xi }\left(t\right)}{\tau_{\text{a}}^{\xi }}+s_{i}^{\xi }\left(t\right)x_{i}^{\xi }\left(t\right)u_{i}^{\xi }\left(t\right)/U_{\text{se}}^{\xi }, \end{array}$$

(4) $$\begin{array}{l} x_{i}^{\xi }\left(t+1\right)=x_{i}^{\xi }\left(t\right)+\frac{1-x_{i}^{\xi }\left(t\right)}{\tau_{\text{R}}^{\xi }}-s_{i}^{\xi }\left(t\right)x_{i}^{\xi }\left(t\right)u_{i}^{\xi }\left(t\right), \end{array}$$

(5) $$\begin{array}{l} u_{i}^{\xi }\left(t+1\right)=u_{i}^{\xi }\left(t\right)+\frac{U_{\text{se}}^{\xi }-u_{i}^{\xi }\left(t\right)}{\tau_{\text{F}}^{\xi }}+U_{\text{se}}^{\xi }\left(1-u_{i}^{\xi }\left(t\right)\right)s_{i}^{\xi }\left(t\right), \end{array}$$

where the ratio $\tau_{\text{R}}^{\xi }/\tau_{\text{F}}^{\xi }$ determines whether the plasticity is short-term depression or facilitation (Wang et al., 2006).

### 2.3. Mean field theory

The dynamic mean field model was developed according to the following two steps: In the first step, a microscopic mean field model is derived from the stochastic model by taking the expectation (noise average), with respect to each variable. This expectation cannot be replaced with the population or time average. In the second step, a macroscopic mean field model is derived from the microscopic model by taking the average over the population. By assuming that the recurrent network is fully connected via synapses with the coupling strength of the order of 1/*N*_ξ_, we obtain an eight-dimensional discrete-time dynamical system, not dependent on the number of neurons, *N*_ξ_.

First, Equations (1) and (2) are converted into the following forms:

(6) $$\begin{array}{l} \langle s_{i}^{\xi }\left(t+1\right)\rangle =g_{\beta^{\xi }}\left[\langle h_{i}^{\xi }\left(t\right)\rangle \right], \end{array}$$

(7) $$\begin{array}{l} \langle h_{i}^{\xi }\left(t\right)\rangle =\overset{N_{\xi }}{\underset{j\neq i}{\sum }}\left[J_{ij}^{\xi \xi }\langle a_{j}^{\xi }\left(t\right)\rangle \right]+\overset{N_{\eta }}{\underset{j=1}{\sum }}\left[J_{ij}^{\xi \eta }\langle a_{j}^{\eta }\left(t\right)\rangle \right]+I^{\xi }, \end{array}$$

where the notation, 〈·〉, indicates the noise average. Similarly, the following equations, corresponding to Equations (3) to (5), are obtained:

(8) $$\begin{array}{l} \langle a_{i}^{\xi }\left(t+1\right)\rangle =\langle a_{i}^{\xi }\left(t\right)\rangle -\frac{\langle a_{i}^{\xi }\left(t\right)\rangle }{\tau_{\text{a}}^{\xi }}+\langle s_{i}^{\xi }\left(t\right)x_{i}^{\xi }\left(t\right)u_{i}^{\xi }\left(t\right)\rangle /U_{\text{se}}^{\xi }, \end{array}$$

(9) $$\begin{array}{l} \langle x_{i}^{\xi }\left(t+1\right)\rangle =\langle x_{i}^{\xi }\left(t\right)\rangle +\frac{1-\langle x_{i}^{\xi }\left(t\right)\rangle }{\tau_{\text{R}}^{\xi }}-\langle s_{i}^{\xi }\left(t\right)x_{i}^{\xi }\left(t\right)u_{i}^{\xi }\left(t\right)\rangle , \end{array}$$

(10) $$\begin{array}{l} \langle u_{i}^{\xi }\left(t+1\right)\rangle =\langle u_{i}^{\xi }\left(t\right)\rangle +\frac{U_{\text{se}}^{\xi }-\langle u_{i}^{\xi }\left(t\right)\rangle }{\tau_{\text{F}}^{\xi }}+U_{\text{se}}^{\xi }\langle \left(1-u_{i}^{\xi }\left(t\right)\right)s_{i}^{\xi }\left(t\right)\rangle . \end{array}$$

Here, we assume that $J_{ij}^{\xi \xi }$ and $J_{ij}^{\xi \eta }$ are of the order of 1/*N*_ξ_ and 1/*N*_η_, respectively; therefore, the correlation between $s_{i}^{\xi }\left(t\right)$ and $x_{i}^{\xi }\left(t\right)$ is negligible when *N*_ξ_ → ∞ (Igarashi et al., 2010). Likewise, the correlation between $s_{i}^{\xi }\left(t\right)$ and $u_{i}^{\xi }\left(t\right)$ approaches zero as *N*_ξ_ → ∞. Furthermore, in a previous study, it has been found that the correlation between $x_{i}^{\xi }\left(t\right)$ and $u_{i}^{\xi }\left(t\right)$ is negligible when the number of neurons is sufficiently large (Katori et al., 2012), while it has been reported that the independence of $x_{i}^{\xi }\left(t\right)$ and $u_{i}^{\xi }\left(t\right)$ is maintained, if there is no facilitation (Tsodyks et al., 1998). Consequently, we assume the following for simplicity:

(11) $$\begin{array}{l} \langle s_{i}^{\xi }\left(t\right)x_{i}^{\xi }\left(t\right)u_{i}^{\xi }\left(t\right)\rangle =\langle s_{i}^{\xi }\left(t\right)\rangle \langle x_{i}^{\xi }\left(t\right)\rangle \langle u_{i}^{\xi }\left(t\right)\rangle , \end{array}$$

(12) $$\begin{array}{l} \langle s_{i}^{\xi }\left(t\right)u_{i}^{\xi }\left(t\right)\rangle =\langle s_{i}^{\xi }\left(t\right)\rangle \langle u_{i}^{\xi }\left(t\right)\rangle . \end{array}$$

By using these relations, we obtain the following microscopic mean field model:

(13) $$\begin{array}{l} m_{i}^{\xi }\left(t+1\right)=g_{\beta^{\xi }}\left[\overset{N_{\xi }}{\underset{j\neq i}{\sum }}\left(J_{ij}^{\xi \xi }A_{j}^{\xi }\left(t\right)\right)+\overset{N_{\eta }}{\underset{j=1}{\sum }}\left(J_{ij}^{\xi \eta }A_{j}^{\eta }\left(t\right)\right)+I^{\xi }\right], \end{array}$$

(14) $$\begin{array}{l} A_{i}^{\xi }\left(t+1\right)=A_{i}^{\xi }\left(t\right)-\frac{A_{i}^{\xi }\left(t\right)}{\tau_{\text{a}}^{\xi }}+m_{i}^{\xi }\left(t\right)X_{i}^{\xi }\left(t\right)U_{i}^{\xi }\left(t\right)/U_{\text{se}}^{\xi }, \end{array}$$

(15) $$\begin{array}{l} X_{i}^{\xi }\left(t+1\right)=X_{i}^{\xi }\left(t\right)+\frac{1-X_{i}^{\xi }\left(t\right)}{\tau_{\text{R}}^{\xi }}-m_{i}^{\xi }\left(t\right)X_{i}^{\xi }\left(t\right)U_{i}^{\xi }\left(t\right), \end{array}$$

(16) $$\begin{array}{l} U_{i}^{\xi }\left(t+1\right)=U_{i}^{\xi }\left(t\right)+\frac{U_{\text{se}}^{\xi }-U_{i}^{\xi }\left(t\right)}{\tau_{\text{F}}^{\xi }}+U_{\text{se}}^{\xi }\left(1-U_{i}^{\xi }\left(t\right)\right)m_{i}^{\xi }\left(t\right), \end{array}$$

where we have set $m_{i}^{\xi }\left(t\right)\equiv \langle s_{i}^{\xi }\left(t\right)\rangle$, $A_{i}^{\xi }\left(t\right)\equiv \langle a_{i}^{\xi }\left(t\right)\rangle$, $X_{i}^{\xi }\left(t\right)\equiv \langle x_{i}^{\xi }\left(t\right)\rangle$, and $U_{i}^{\xi }\left(t\right)\equiv \langle u_{i}^{\xi }\left(t\right)\rangle$, respectively.

We represent the fixed point of the microscopic mean field model as ${\bar{m}}_{i}^{\xi }$, ${Ā}_{i}^{\xi }$, ${\bar{X}}_{i}^{\xi }$, and ${Ū}_{i}^{\xi }$. The fixed point for Equations (14) to (16) is obtained as follows:

(17) $$\begin{array}{l} {Ā}_{i}^{\xi }=\frac{\tau_{\text{a}}^{\xi }{Ū}_{i}^{\xi }{\bar{m}}_{i}^{\xi }{\bar{X}}_{i}^{\xi }}{U_{\text{se}}^{\xi }}, \end{array}$$

(18) $$\begin{array}{l} {\bar{X}}_{i}^{\xi }=\frac{1}{1+\tau_{\text{R}}^{\xi }{Ū}_{i}^{\xi }{\bar{m}}_{i}^{\xi }}, \end{array}$$

(19) $$\begin{array}{l} {Ū}_{i}^{\xi }=\frac{U_{\text{se}}^{\xi }\left(1+\tau_{\text{F}}^{\xi }{\bar{m}}_{i}^{\xi }\right)}{1+\tau_{\text{F}}^{\xi }U_{\text{se}}^{\xi }{\bar{m}}_{i}^{\xi }}. \end{array}$$

Using these equations, we obtain the value ${\bar{m}}_{i}^{\xi }$ at the fixed point as follows:

(20) $$\begin{array}{l} {\bar{m}}_{i}^{\xi }=g_{\beta^{\xi }}[\sum_{j\;\neq \;i}^{N_{\xi }}J_{ij}^{\xi \xi }\left(\frac{\tau_{\text{a}}^{\xi }{\bar{m}}_{j}^{\xi }(1+\tau_{\text{F}}^{\xi }{\bar{m}}_{j}^{\xi })}{1+(\tau_{\text{F}}^{\xi }+\tau_{\text{R}}^{\xi })U_{\text{se}}^{\xi }{\bar{m}}_{j}^{\xi }+U_{\text{se}}^{\xi }\tau_{\text{F}}^{\xi }\tau_{\text{R}}^{\xi }{\bar{m}}_{j}^{\xi }{\bar{m}}_{j}^{\xi }}\right)\\ \;+\;\sum_{j\;=\;1}^{N_{\eta }}J_{ij}^{\xi \eta }\left(\frac{\tau_{\text{a}}^{\eta }{\bar{m}}_{j}^{\eta }(1+\tau_{\text{F}}^{\eta }{\bar{m}}_{j}^{\eta })}{1+(\tau_{\text{F}}^{\eta }+\tau_{\text{R}}^{\eta })U_{\text{se}}^{\eta }{\bar{m}}_{j}^{\eta }+U_{\text{se}}^{\eta }\tau_{\text{F}}^{\eta }\tau_{\text{R}}^{\eta }{\bar{m}}_{j}^{\eta }{\bar{m}}_{j}^{\eta }}\right)+I^{\xi }]. \end{array}$$

We derive a macroscopic mean field model by considering a network with all-to-all connections, where the weights $J_{ij}^{\xi \xi }$ and $J_{ij}^{\xi \eta }$ are given as follows:

(21) $$\begin{array}{l} J_{ij}^{\xi \xi }=\frac{J_{0}^{\xi \xi }}{N_{\xi }}, \end{array}$$

(22) $$\begin{array}{l} J_{ij}^{\xi \eta }=\frac{J_{0}^{\xi \eta }}{N_{\eta }}. \end{array}$$

Here, $J_{0}^{\xi \xi }$ and $J_{0}^{\xi \eta }$ are the parameters specifying the strength of the uniform connections. Because of this synaptic connection uniformity, the variables $m_{i}^{\xi }$, $A_{i}^{\xi }$, $X_{i}^{\xi }$, and $U_{i}^{\xi }$ can be substituted with their respective population averages $m_{0}^{\xi }=\left(1/N_{\xi }\right)\overset{N_{\xi }}{\underset{i=1}{\sum }}m_{i}^{\xi }$, $A_{0}^{\xi }=\left(1/N_{\xi }\right)\overset{N_{\xi }}{\underset{i=1}{\sum }}A_{i}^{\xi }$, $X_{0}^{\xi }=\left(1/N_{\xi }\right)\overset{N_{\xi }}{\underset{i=1}{\sum }}X_{i}^{\xi }$, and $U_{0}^{\xi }=\left(1/N_{\xi }\right)\overset{N_{\xi }}{\underset{i=1}{\sum }}U_{i}^{\xi }$. The macroscopic mean field model for a network with uniform connections is given as follows:

(23) $$\begin{array}{l} m_{0}^{\xi }\left(t+1\right)=F_{m}^{\xi }\left(\Omega \left(t\right)\right), \end{array}$$

(24) $$\begin{array}{l} A_{0}^{\xi }\left(t+1\right)=F_{A}^{\xi }\left(\Omega \left(t\right)\right), \end{array}$$

(25) $$\begin{array}{l} X_{0}^{\xi }\left(t+1\right)=F_{X}^{\xi }\left(\Omega \left(t\right)\right), \end{array}$$

(26) $$\begin{array}{l} U_{0}^{\xi }\left(t+1\right)=F_{U}^{\xi }\left(\Omega \left(t\right)\right), \end{array}$$

where

(27) $$\begin{array}{l} F_{m}^{\xi }\left(\Omega \left(t\right)\right)=g_{\beta^{\xi }}\left[J_{0}^{\xi \xi }A_{0}^{\xi }+J_{0}^{\xi \eta }A_{0}^{\eta }+I^{\xi }\right], \end{array}$$

(28) $$\begin{array}{l} F_{A}^{\xi }\left(\Omega \left(t\right)\right)=A_{0}^{\xi }-\frac{A_{0}^{\xi }}{\tau_{\text{a}}^{\xi }}+m_{0}^{\xi }X_{0}^{\xi }U_{0}^{\xi }/U_{\text{se}}^{\xi }, \end{array}$$

(29) $$\begin{array}{l} F_{X}^{\xi }\left(\Omega \left(t\right)\right)=X_{0}^{\xi }+\frac{1-X_{0}^{\xi }}{\tau_{\text{R}}^{\xi }}-m_{0}^{\xi }X_{0}^{\xi }U_{0}^{\xi }, \end{array}$$

(30) $$\begin{array}{l} F_{U}^{\xi }\left(\Omega \left(t\right)\right)=U_{0}^{\xi }+\frac{U_{\text{se}}^{\xi }-U_{0}^{\xi }}{\tau_{\text{F}}^{\xi }}+U_{\text{se}}^{\xi }\left(1-U_{0}^{\xi }\right)m_{0}^{\xi }, \end{array}$$

with the state vector Ω(*t*) defined as follows:

(31) $$\begin{array}{l} \Omega \left(t\right)={\left[m_{0}^{\text{E}}\left(t\right),m_{0}^{\text{I}}\left(t\right),A_{0}^{\text{E}}\left(t\right),A_{0}^{\text{I}}\left(t\right),X_{0}^{\text{E}}\left(t\right),X_{0}^{\text{I}}\left(t\right),U_{0}^{\text{E}}\left(t\right),U_{0}^{\text{I}}\left(t\right)\right]}^{\text{T}}. \end{array}$$

As shown in the Results section, the dynamic mean field model is consistent with the simulation on the stochastic model.

By modifying Equation (20), the fixed point for the macroscopic mean field model can be calculated as follows:

(32) $$\begin{array}{l} {\bar{m}}^{\text{E}}=f_{\text{E}}\left({\bar{m}}^{\text{E}},{\bar{m}}^{\text{I}}\right), \end{array}$$

(33) $$\begin{array}{l} {\bar{m}}^{\text{I}}=f_{\text{I}}\left({\bar{m}}^{\text{I}},{\bar{m}}^{\text{E}}\right), \end{array}$$

where

(34) $$\begin{array}{l} f_{\xi }({\bar{m}}^{\xi },{\bar{m}}^{\eta })\\ =\;g_{\beta^{\xi }}[J_{0}^{\xi \xi }\left(\frac{\tau_{\text{a}}^{\xi }{\bar{m}}^{\xi }\left(1+\tau_{\text{F}}^{\xi }{\bar{m}}^{\xi }\right)}{1+\left(\tau_{\text{F}}^{\xi }+\tau_{\text{R}}^{\xi }\right)U_{\text{se}}^{\xi }{\bar{m}}^{\xi }+U_{\text{se}}^{\xi }\tau_{\text{F}}^{\xi }\tau_{\text{R}}^{\xi }{\bar{m}}^{\xi }{\bar{m}}^{\xi }}\right)\\ +J_{0}^{\xi \eta }\left(\frac{\tau_{\text{a}}^{\eta }{\bar{m}}^{\eta }\left(1+\tau_{\text{F}}^{\eta }{\bar{m}}^{\eta }\right)}{1+\left(\tau_{\text{F}}^{\eta }+\tau_{\text{R}}^{\eta }\right)U_{\text{se}}^{\eta }{\bar{m}}^{\eta }+U_{\text{se}}^{\eta }\tau_{\text{F}}^{\eta }\tau_{\text{R}}^{\eta }{\bar{m}}^{\eta }{\bar{m}}^{\eta }}\right)+I^{\xi }]. \end{array}$$

After solving the above equations simultaneously, we obtain the values ${\bar{m}}_{0}^{\text{E}}$ and ${\bar{m}}_{0}^{\text{I}}$ at the fixed point. By substituting ${\bar{m}}_{0}^{\text{E}}$ and ${\bar{m}}_{0}^{\text{I}}$ into the following fixed point equations,

(35) $$\begin{array}{l} {Ā}_{0}^{\xi }=\frac{\tau_{\text{a}}^{\xi }{Ū}_{0}^{\xi }{\bar{m}}_{0}^{\xi }{\bar{X}}_{0}^{\xi }}{U_{\text{se}}^{\xi }}, \end{array}$$

(36) $$\begin{array}{l} {\bar{X}}_{0}^{\xi }=\frac{1}{1+\tau_{\text{R}}^{\xi }{Ū}_{0}^{\xi }{\bar{m}}_{0}^{\xi }}, \end{array}$$

(37) $$\begin{array}{l} {Ū}_{0}^{\xi }=\frac{U_{\text{se}}^{\xi }\left(1+\tau_{\text{F}}^{\xi }{\bar{m}}_{0}^{\xi }\right)}{1+\tau_{\text{F}}^{\xi }U_{\text{se}}^{\xi }{\bar{m}}_{0}^{\xi }}, \end{array}$$

we obtain the values ${Ā}_{0}^{\xi }$, ${\bar{X}}_{0}^{\xi }$, and ${Ū}_{0}^{\xi }$ at the fixed point. Because nonlinear simultaneous equations cannot generally be solved analytically due to the function $g_{\beta^{\xi }}\left[\cdot \right]$, we use Newton's method to numerically obtain the fixed point in the Results section.

We analyze the stability of the fixed point with small deviations, $\delta m_{0}^{\xi }\left(t\right)$, $\delta A_{0}^{\xi }\left(t\right)$, $\delta X_{0}^{\xi }\left(t\right)$, and $\delta U_{0}^{\xi }\left(t\right)$, around the fixed point as follows:

(38) $$\begin{array}{l} m_{0}^{\xi }\left(t\right)={\bar{m}}_{0}^{\xi }+\delta m_{0}^{\xi }\left(t\right), \end{array}$$

(39) $$\begin{array}{l} A_{0}^{\xi }\left(t\right)={Ā}_{0}^{\xi }+\delta A_{0}^{\xi }\left(t\right), \end{array}$$

(40) $$\begin{array}{l} X_{0}^{\xi }\left(t\right)={\bar{X}}_{0}^{\xi }+\delta X_{0}^{\xi }\left(t\right), \end{array}$$

(41) $$\begin{array}{l} U_{0}^{\xi }\left(t\right)={Ū}_{0}^{\xi }+\delta U_{0}^{\xi }\left(t\right). \end{array}$$

By neglecting the higher order components, we obtain the following locally linearized equation:

(42) $$\begin{array}{l} \left(\begin{array}{c} \delta m_{0}^{\text{E}}\left(t+1\right)\\ \delta A_{0}^{\text{E}}\left(t+1\right)\\ \delta X_{0}^{\text{E}}\left(t+1\right)\\ \delta U_{0}^{\text{E}}\left(t+1\right)\\ \delta m_{0}^{\text{I}}\left(t+1\right)\\ \delta A_{0}^{\text{I}}\left(t+1\right)\\ \delta X_{0}^{\text{I}}\left(t+1\right)\\ \delta U_{0}^{\text{I}}\left(t+1\right) \end{array}\right)=K\left(\begin{array}{c} \delta m_{0}^{\text{E}}\left(t\right)\\ \delta A_{0}^{\text{E}}\left(t\right)\\ \delta X_{0}^{\text{E}}\left(t\right)\\ \delta U_{0}^{\text{E}}\left(t\right)\\ \delta m_{0}^{\text{I}}\left(t\right)\\ \delta A_{0}^{\text{I}}\left(t\right)\\ \delta X_{0}^{\text{I}}\left(t\right)\\ \delta U_{0}^{\text{I}}\left(t\right) \end{array}\right), \end{array}$$

where *K* denotes the following Jacobian matrix:

(43) $$\begin{array}{l} K=\left(\begin{array}{cccccccc} K_{mm}^{\text{EE}} & K_{mA}^{\text{EE}} & K_{mX}^{\text{EE}} & K_{mU}^{\text{EE}} & K_{mm}^{\text{EI}} & K_{mA}^{\text{EI}} & K_{mX}^{\text{EI}} & K_{mU}^{\text{EI}}\\ K_{Am}^{\text{EE}} & K_{AA}^{\text{EE}} & K_{AX}^{\text{EE}} & K_{AU}^{\text{EE}} & K_{Am}^{\text{EI}} & K_{AA}^{\text{EI}} & K_{AX}^{\text{EI}} & K_{AU}^{\text{EI}}\\ K_{Xm}^{\text{EE}} & K_{XA}^{\text{EE}} & K_{XX}^{\text{EE}} & K_{XU}^{\text{EE}} & K_{Xm}^{\text{EI}} & K_{XA}^{\text{EI}} & K_{XX}^{\text{EI}} & K_{XU}^{\text{EI}}\\ K_{Um}^{\text{EE}} & K_{UA}^{\text{EE}} & K_{UX}^{\text{EE}} & K_{UU}^{\text{EE}} & K_{Um}^{\text{EI}} & K_{UA}^{\text{EI}} & K_{UX}^{\text{EI}} & K_{UU}^{\text{EI}}\\ K_{mm}^{\text{IE}} & K_{mA}^{\text{IE}} & K_{mX}^{\text{IE}} & K_{mU}^{\text{IE}} & K_{mm}^{\text{II}} & K_{mA}^{\text{II}} & K_{mX}^{\text{II}} & K_{mU}^{\text{II}}\\ K_{Am}^{\text{IE}} & K_{AA}^{\text{IE}} & K_{AX}^{\text{IE}} & K_{AU}^{\text{IE}} & K_{Am}^{\text{II}} & K_{AA}^{\text{II}} & K_{AX}^{\text{II}} & K_{AU}^{\text{II}}\\ K_{Xm}^{\text{IE}} & K_{XA}^{\text{IE}} & K_{XX}^{\text{IE}} & K_{XU}^{\text{IE}} & K_{Xm}^{\text{II}} & K_{XA}^{\text{II}} & K_{XX}^{\text{II}} & K_{XU}^{\text{II}}\\ K_{Um}^{\text{IE}} & K_{UA}^{\text{IE}} & K_{UX}^{\text{IE}} & K_{UU}^{\text{IE}} & K_{Um}^{\text{II}} & K_{UA}^{\text{II}} & K_{UX}^{\text{II}} & K_{UU}^{\text{II}} \end{array}\right), \end{array}$$

and each element of this matrix is given as follows:

(44) $$\begin{array}{l} K_{mA}^{\xi \xi }=\frac{\partial F_{m}^{\xi }}{\partial A^{\xi }}=g_{\beta^{\xi }}^{\prime }\left[h^{\xi }\right]J_{0}^{\xi \xi }, \end{array}$$

(45) $$\begin{array}{l} K_{mA}^{\xi \eta }=\frac{\partial F_{m}^{\xi }}{\partial A^{\eta }}=g_{\beta^{\xi }}^{\prime }\left[h^{\xi }\right]J_{0}^{\xi \eta }, \end{array}$$

where

(46) $$\begin{array}{l} g_{\beta^{\xi }}^{\prime }\left[h^{\xi }\right]=\frac{\beta^{\xi }}{2}\left[1-\tanh^{2}\left(\beta^{\xi }h^{\xi }\right)\right], \end{array}$$

(47) $$\begin{array}{l} h^{\xi }=J_{0}^{\xi \xi }A^{\xi }+J_{0}^{\xi \eta }A^{\eta }+I^{\xi }. \end{array}$$

Furthermore, the remaining elements of matrix *K* are given as follows:

(48) $$\begin{array}{l} K_{Am}^{\xi \xi }=\frac{U^{\xi }X^{\xi }}{U_{\text{se}}^{\xi }}, \end{array}$$

(49) $$\begin{array}{l} K_{AX}^{\xi \xi }=\frac{m^{\xi }U^{\xi }}{U_{\text{se}}^{\xi }}, \end{array}$$

(50) $$\begin{array}{l} K_{AU}^{\xi \xi }=\frac{m^{\xi }X^{\xi }}{U_{\text{se}}^{\xi }}, \end{array}$$

(51) $$\begin{array}{l} K_{AA}^{\xi \xi }=1-\frac{1}{\tau_{\text{a}}^{\xi }}, \end{array}$$

(52) $$\begin{array}{l} K_{Xm}^{\xi \xi }=-U^{\xi }X^{\xi }, \end{array}$$

(53) $$\begin{array}{l} K_{XX}^{\xi \xi }=\left(1-\frac{1}{\tau_{\text{R}}^{\xi }}\right)-m^{\xi }U^{\xi }, \end{array}$$

(54) $$\begin{array}{l} K_{XU}^{\xi \xi }=-m^{\xi }X^{\xi }, \end{array}$$

(55) $$\begin{array}{l} K_{Um}^{\xi \xi }=U_{\text{se}}^{\xi }\left(1-U^{\xi }\right), \end{array}$$

(56) $$\begin{array}{l} K_{UU}^{\xi \xi }=\left(1-\frac{1}{\tau_{\text{F}}^{\xi }}\right)-U_{\text{se}}^{\xi }m^{\xi }, \end{array}$$

and other elements are zeroes. We numerically analyze the stability of the fixed point by eigenvalue analysis with this Jacobian matrix.

### 2.4. Slice analysis

**Figure 2:**
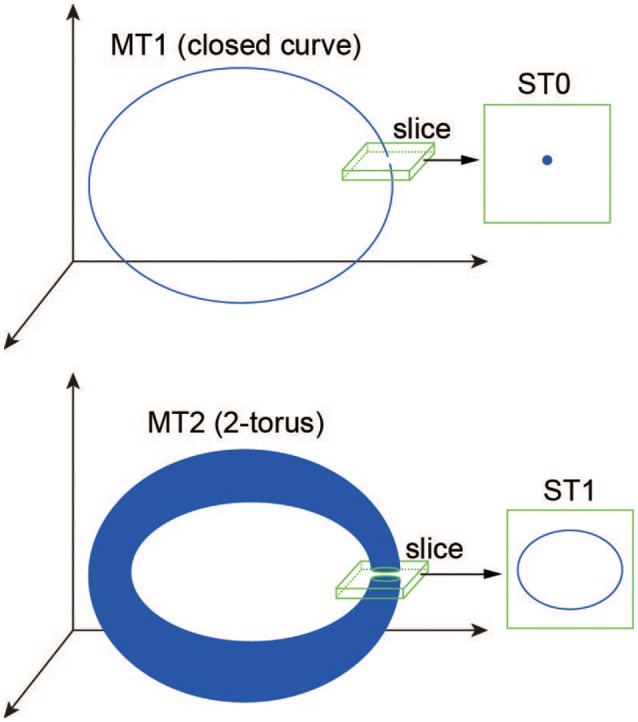
**A schematic definition of the ST0 and ST1 converted from the MT1 and MT2 via the slice (Komuro et al., **2016**)**.

We use the slice analysis developed by Komuro et al. (2016), to elucidate the bifurcations of quasi-periodic oscillations arising from the proposed model. Mathematically, a slice with a width of ϵ is defined here as

(57) $$\begin{array}{l} \Sigma^{\epsilon }=\left\{\Omega \in {\mathbb{R}}^{8}|\text{dist}\left(\Omega ,\Sigma \right)<\epsilon \right\}, \end{array}$$

where dist(·, ·) denotes the Euclidean distance between a point of the state vector Ω(*t*) (Equation 31) and a codimension-one plane Σ, called “section” (Komuro et al., 2016). Here, to differentiate a trajectory in the state space from one in the section qualitatively, we introduce the following two useful terms: a *d*-dimensional torus in map (MT*d*) and a *d*-dimensional torus in section (ST*d*) (Kamiyama et al., 2014; Komuro et al., 2016). Accordingly, for example, an MT1 (a closed curve) and an MT2 (a two-dimensional torus) are converted into an ST0 (an isolated point) and an ST1 (a closed curve), respectively, via the slice (Figure 2). Because the bifurcation of MT*d* can be interpreted as that of ST(*d* − 1) almost equivalently, we apply the conventional bifurcation theory to ST*d* in order to consider its bifurcations as well as those of MT*d* (Komuro et al., 2016).

## 3. Results

In this section, we analyze a variety of bifurcation structures arising from the proposed model by considering $\tau_{\text{a}}^{\xi }$, $J_{0}^{\xi \xi }$, $J_{0}^{\xi \eta }$, *I*^ξ^, and $\tau_{\text{R}}^{\xi }/\tau_{\text{F}}^{\xi }$ to be bifurcation parameters and *T*^ξ^ and $U_{\text{se}}^{\xi }$ to be constants; we set *T*^ξ^ = 0.8 and $U_{\text{se}}^{\xi }=0.1$, respectively, because the activation function $g_{\beta^{\xi }}\left[\cdot \right]$ used here is an idealized sigmoid form different from the experimentally known frequency-current relationship (Tateno et al., 2004) and $U_{\text{se}}^{\xi }$ merely determines the steady value of the calcium concentration. The bifurcation analysis focuses on two types of dynamic synapses, namely short-term depression ($\tau_{\text{R}}^{\xi }/\tau_{\text{F}}^{\xi }\gg 1$) and facilitation ($\tau_{\text{R}}^{\xi }/\tau_{\text{F}}^{\xi }\ll 1$) synapses, where $\tau_{\text{R}}^{\xi }$ has been fixed at 70 and $\tau_{\text{F}}^{\xi }$ ranges between 1 and 140 for the sake of simplicity. In this study, first, we individually investigate an excitatory and an inhibitory networks, by setting $J_{0}^{\text{EI}}=0$ and $J_{0}^{\text{EE}}\geq 0$ for the excitatory network and $J_{0}^{\text{IE}}=0$ and $J_{0}^{\text{II}}<0$ for the inhibitory network. Next, we analyze a network composed of both the excitatory and the inhibitory subnetworks. Throughout the study, when the stochastic model is simulated to be compared with the corresponding macroscopic mean field model, we use $N_{\xi }=10^{4}$. In the following, we omit the superscripts attached to the variables and the parameters for the sake of simplicity when examining the aforementioned two networks independently.

**Figure 3:**
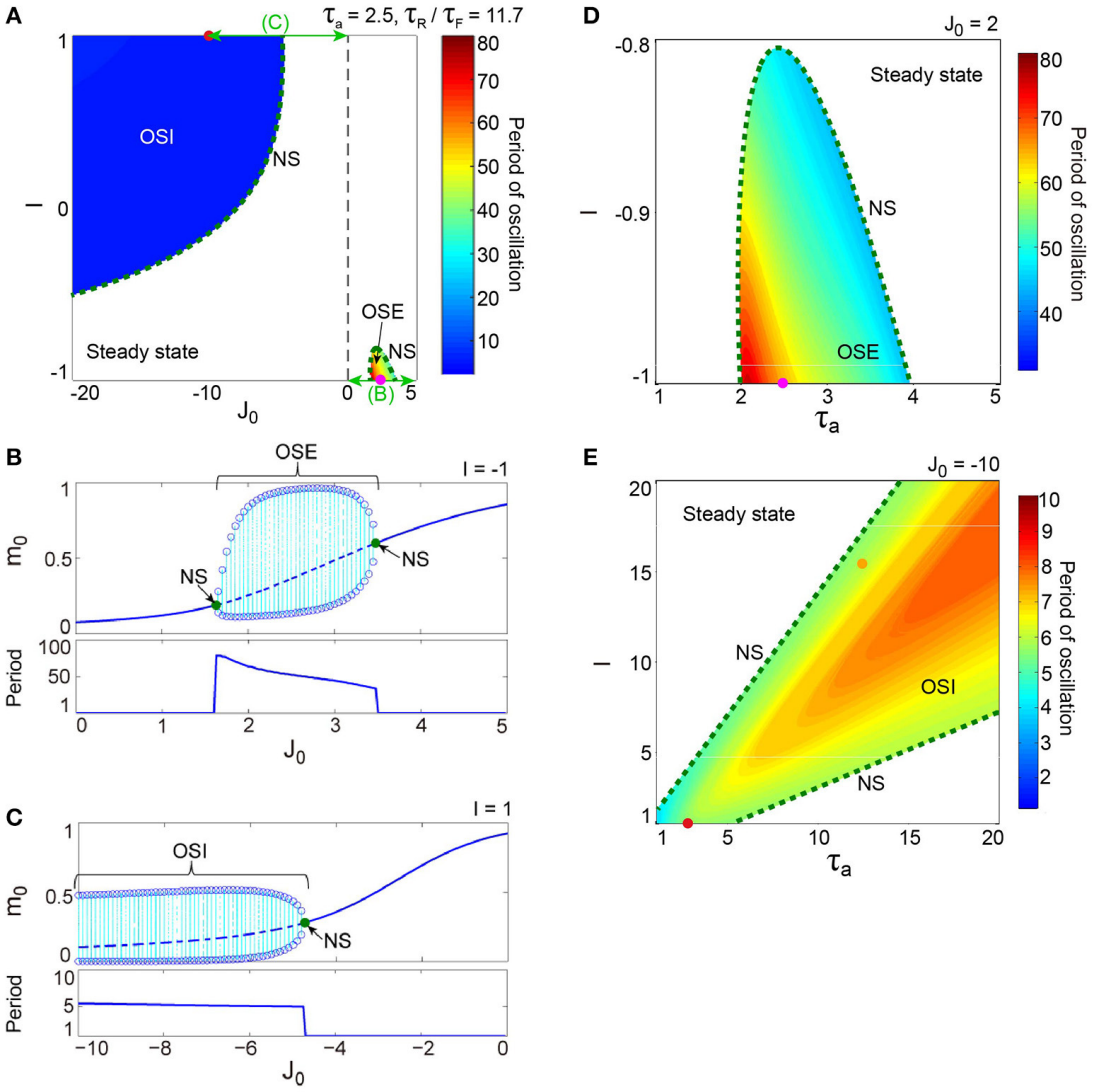
**A dynamical qualitative change between the excitatory and the inhibitory networks with dynamic synapses**. **(A)** (*J*_0_, *I*) phase diagram. The OSE and the OSI regions appear from the steady state region via the NS bifurcation set. The oscillation on the OSI region is faster than that on the OSE region. **(B)** The bifurcation diagram and the corresponding oscillatory period, with respect to the non-negative coupling strength (*J*_0_ ≥ 0). A set of stable fixed points is indicated by the solid curve, while a set of unstable fixed points is indicated by the dashed curve. The OSE state is indicated by the dotted orbits with open circles, which represent their maximal and minimal values. **(C)** The bifurcation diagram and the corresponding oscillatory period, with respect to the negative coupling strength (*J*_0_ < 0). In **(B,C)**, the steady state, and the OSE or the OSI state, are exchanged via the NS bifurcation point, indicated by the filled circles. **(D)** (τ_a_, *I*) phase diagram for the OSE state. **(E)** (τ_a_, *I*) phase diagram for the OSI state. The oscillatory period in both the OSE and the OSI regions changes depending on τ_a_ and *I*.

**Figure 4:**
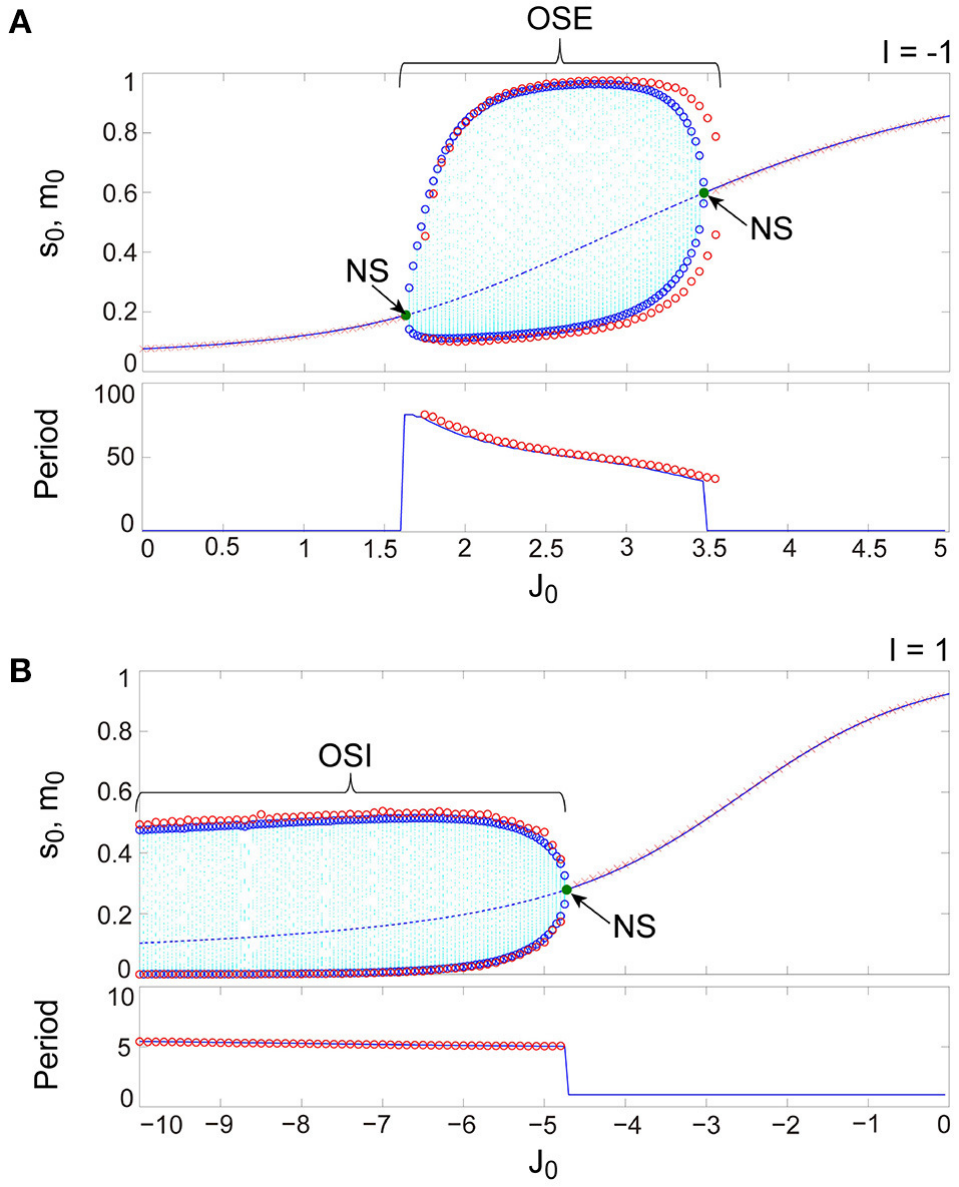
**A comparison between typical bifurcation diagrams generated from the stochastic (red) and the macroscopic (blue) mean field models**. **(A)** The bifurcation diagram and the corresponding oscillatory period, with respect to the non-negative coupling strength (*J*_0_ ≥ 0). **(B)** The bifurcation diagram and the corresponding oscillatory period, with respect to the negative coupling strength (*J*_0_ < 0). The formats of **(A,B)** for the macroscopic mean field model are the same as those in Figures 3B,C, respectively. The red open circles indicate maximal and minimal values of the OSE state for **(A)**, those of the OSI state for (B), and the oscillatory period for these states. A pair of red open circles, corresponding to maximal and minimal values, were plotted when the absolute difference between these values exceeded a certain threshold value. The red cross indicates the average value of the stochastic variable *s*_0_(*t*) over realization. The macroscopic mean field model shows good agreement with the stochastic model in terms of both the distribution of trajectories and the oscillatory period.

**Figure 5:**
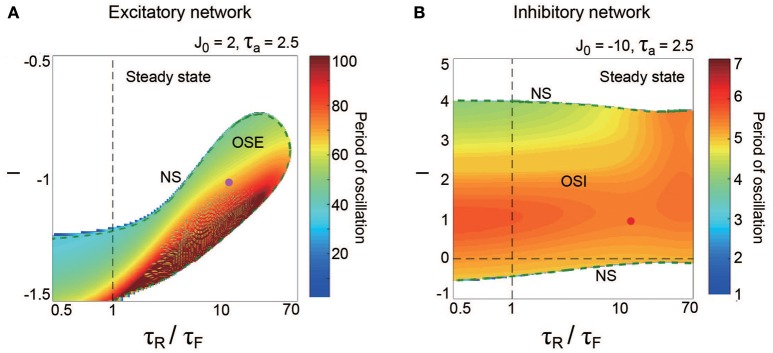
**Effect of dynamic synapse properties on oscillatory phenomena generated from the excitatory network for (A)** and from the inhibitory network for **(B)**. The horizontal axes for both panels are displayed in the logarithmic scale. **(A)** (τ_R_/τ_F_, *I*) diagram for the OSE state. The OSE region is generated from the steady state via the NS bifurcation set. **(B)** (τ_R_/τ_F_, *I*) diagram for the OSI state. The OSI region is generated from the steady state via the two NS bifurcation sets. For both the OSE and the OSI regions, the oscillatory period changes depending on τ_R_/τ_F_ and input *I*.

**Figure 6:**
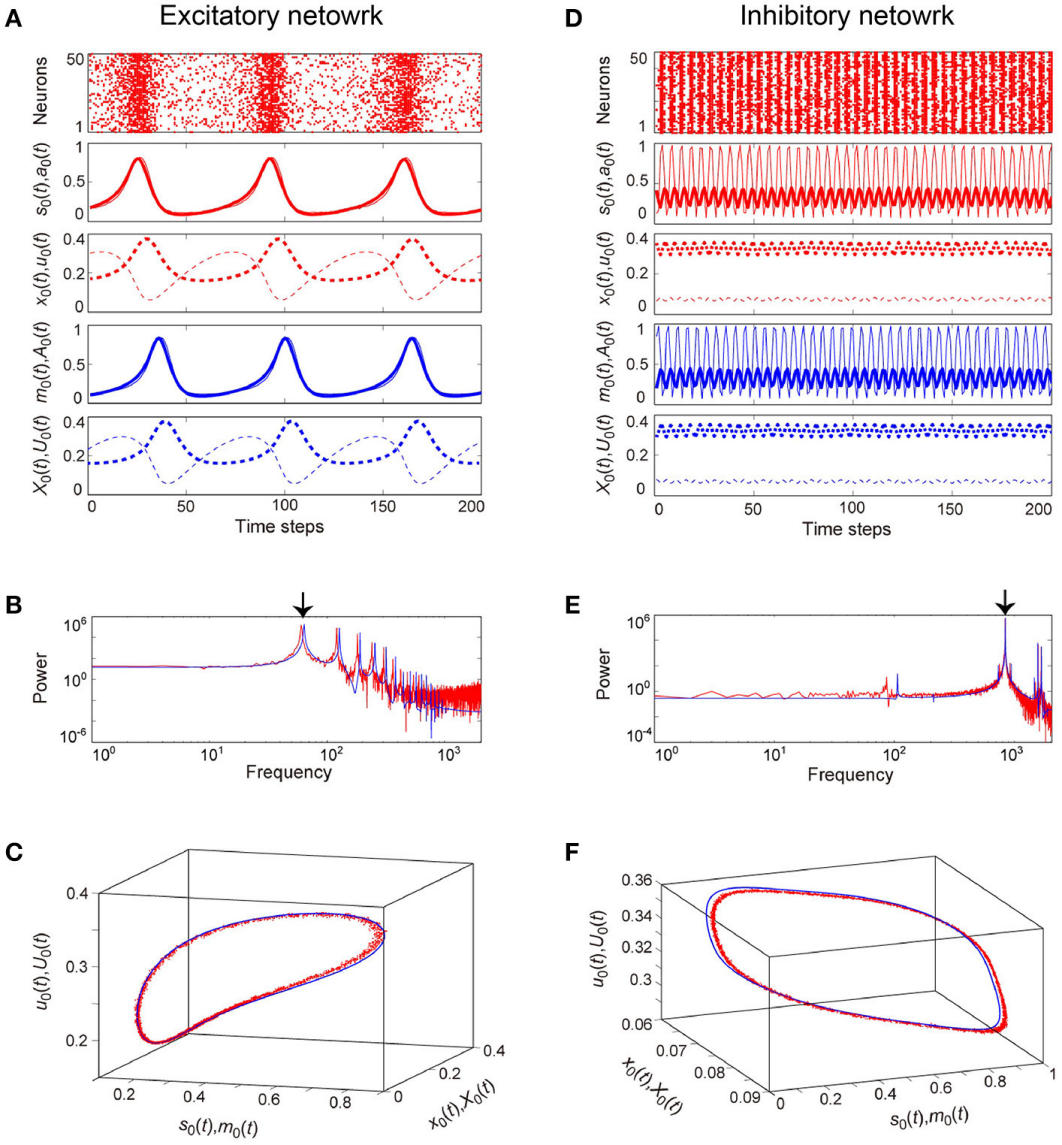
**Examples of dynamics emergent from the excitatory and the inhibitory networks reflecting properties of dynamic synapses**. The following parameter sets were used: (*J*_0_, *I*, τ_a_) = (2, −1, 2.5) for the excitatory network, (*J*_0_, *I*, τ_a_) = (−10, 1, 12.5) for the inhibitory network, and τ_R_/τ_F_ = 11.7 for the dynamic synapses. The dynamics generated from the stochastic and the macroscopic mean field models are indicated by the red and the blue colors, respectively. **(A,D)** The raster plots and the time courses of variables included in the stochastic and the macroscopic mean field models. The dots indicate 50 of 10^4^ excitatory or inhibitory neurons where *s*_*i*_(*t*) = 1. The stochastic variables, *s*_0_(*t*), *a*_0_(*t*), *x*_0_(*t*), and *u*_0_(*t*) and the mean field variables, *m*_0_(*t*), *A*_0_(*t*), *X*_0_(*t*), and *U*_0_(*t*) are displayed as time courses in the thin solid, the thick solid, the thin dashed, and the thick dashed lines, respectively. The time courses of *s*_0_(*t*) and *a*_0_(*t*), and those of *m*_0_(*t*) and *A*_0_(*t*) for **(A)** are almost overlapping. **(B,E)** The power spectra of variables *s*_0_(*t*) and *m*_0_(*t*). The two arrows indicate the fundamental frequency components. **(C,F)** The closed curves in the state space. The dynamics of the excitatory network exhibits relatively slow oscillations (the left side), while that of the inhibitory network shows fast oscillations (the right side).

**Figure 7:**
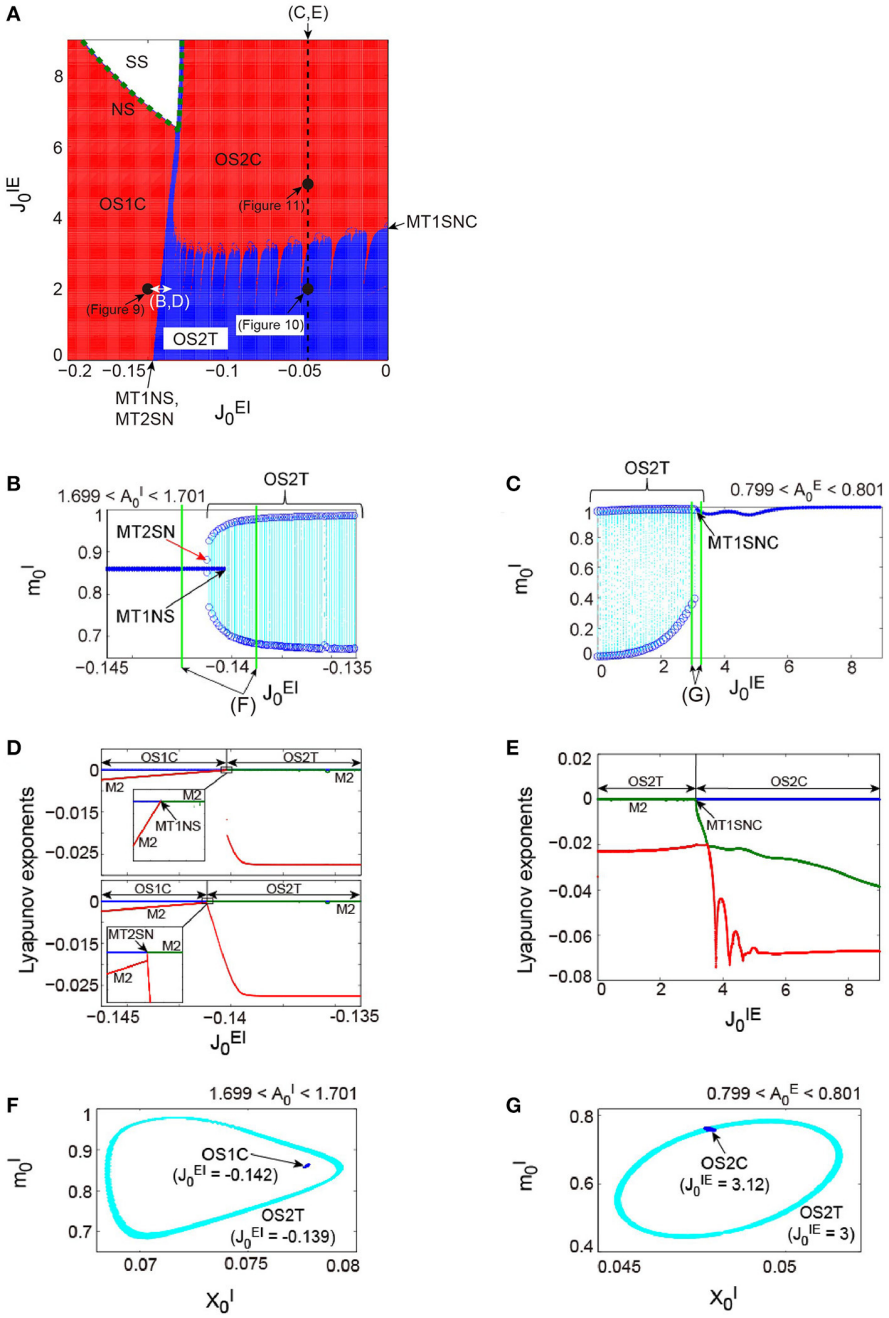
**The bifurcation structure, emergent from the network composed of the excitatory and the inhibitory subnetworks reflecting properties of dynamic synapses**. The parameters used for these subnetworks were the same as those for the individual excitatory network and the individual inhibitory one in Figure 6, respectively. **(A)** $\left(J_{0}^{\text{EI}},J_{0}^{\text{IE}}\right)$ phase diagram. The number of zero-Lyapunov exponents is displayed according to the following manner: 0, 1, and 2 correspond to the white, red, and blue colors, respectively. Both the OS1C and the OS2C regions are generated from the SS region via the NS bifurcation set, while the OS2T region is generated via the following two types of bifurcation: the MT1NS bifurcation from the OS1C region and the MT1SNC bifurcation from the OS2C region (Kamiyama et al., 2014; Komuro et al., 2016). There exists a hysteresis area between the OS1C and the OS2T regions with a very narrow size such that the MT2SN bifurcation intermediates between these regions (Komuro et al., 2016). **(B)** The bifurcation diagram of trajectories in the slice with respect to $J_{0}^{\text{EI}}$, where section $A_{0}^{\text{I}}=1.7$ with width ϵ = 0.001 was used. A set of the ST0 for the OS1C state is indicated by the solid curve, while the ST1 for the OS2T state is indicated by the dotted orbits with open circles which represent their maximal and minimal values. The OS2T state is generated from the OS1C state via the MT1NS bifurcation, whereas the OS1C state is generated from the OS2T state via the MT2SN bifurcation (Kamiyama et al., 2014; Komuro et al., 2016). **(C)** The bifurcation diagram of trajectories in the slice, with respect to $J_{0}^{\text{IE}}$, where section $A_{0}^{\text{E}}=0.8$ with width ϵ = 0.001 was used. The OS2T state is generated from the OS2C state via the MT1SNC bifurcation (Komuro et al., 2016). **(D)** The bifurcation diagrams of the Lyapunov exponents, with respect to $J_{0}^{\text{EI}}$. The exponents for the upper diagram were calculated with an increase in $J_{0}^{\text{EI}}$, whereas, those for the lower diagram were calculated with a decrease in $J_{0}^{\text{EI}}$. **(E)** The bifurcation diagram of the Lyapunov exponents, with respect to $J_{0}^{\text{IE}}$. For **(D,E)**, the first, second, and third exponents are displayed in the blue, green, and red colors, respectively, and exponents with multiplicity of two are indicated by the notation “M2.” **(F)** The slice, applied to both the MT1 and the MT2 near the MT1NS bifurcation point, where section $A_{0}^{\text{I}}=1.7$ with width ϵ = 0.001 was used. **(G)** The slice, applied to both the MT1 and the MT2 near the MT1SNC bifurcation point, where section $A_{0}^{\text{E}}=0.8$ with width ϵ = 0.001 was used. For **(F,G)**, the trajectories in the slice applied to the MT1 and MT2 are displayed in the blue and cyan colors, respectively. Although the ST1 (MT2) appears separated from the ST0 (MT1) when the ST0NS (MT1NS) bifurcation occurs, the ST1 (MT2) is formed on the ST0 (MT1) when the ST0SNC (MT1SNC) bifurcation occurs (Komuro et al., 2016).

**Figure 8:**
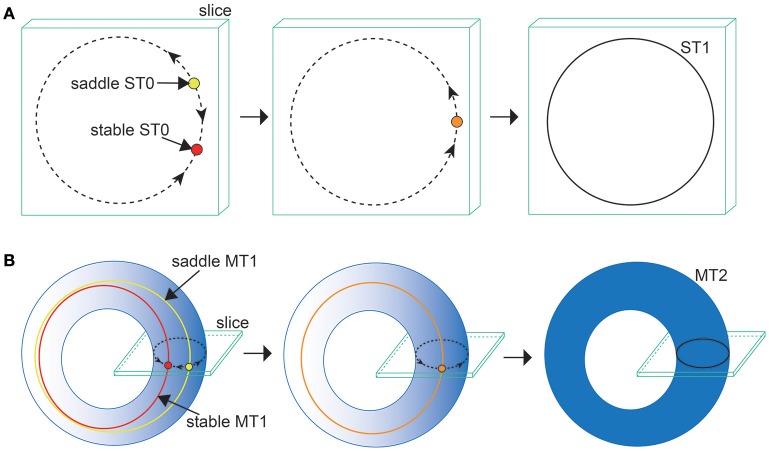
**Illustrations of (A)** the ST0SNC and **(B)** the MT1SNC bifurcations (Komuro et al., 2016). Before the bifurcation occurs, the unstable set of a saddle ST0 (a saddle MT1) generates a one-dimensional torus (a two-dimensional torus), indicated by the dotted curve for **(A)** (the gradation area for **B**). Via the ST0SNC (MT1SNC) bifurcation, the unstable set is stabilized, and accordingly, an ST1 (an MT2) appears, indicated by the solid curve for **(A)** (the uniformly filled area for **B**).

**Figure 9:**
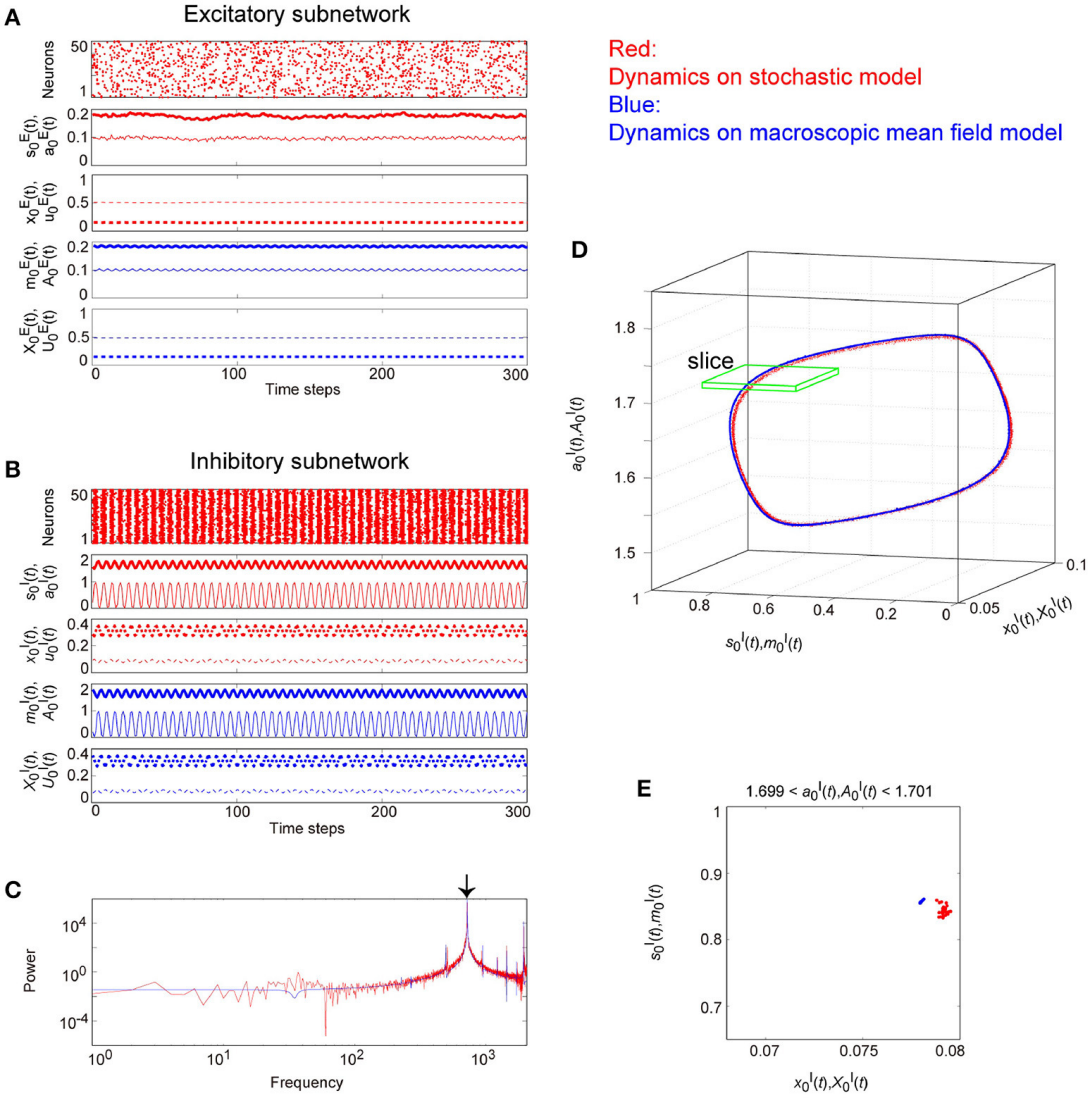
**The typical dynamics emergent from the OS1C state in the network with dynamic synapses**. The following parameter sets were used: $\left(J_{0}^{\text{EE}},I^{\text{E}},\tau_{\text{a}}^{\text{E}}\right)=\left(2,-1,2.5\right)$ for the excitatory subnetwork, $\left(J_{0}^{\text{II}},I^{\text{I}},\tau_{\text{a}}^{\text{I}}\right)=\left(-10,16,12.5\right)$ for the inhibitory subnetwork, $\left(J_{0}^{\text{EI}},J_{0}^{\text{IE}}\right)=\left(-0.15,2\right)$ for the bidirectional coupling between these subnetworks, and $\tau_{\text{R}}^{\xi }/\tau_{\text{F}}^{\xi }=11.7$ for the dynamic synapses. **(A,B)** The raster plots and the time courses of variables included in the stochastic and the macroscopic mean field models. The dots indicate 50 of 10^4^ excitatory or inhibitory neurons where $s_{i}^{\text{E}}\left(t\right)=1$ and $s_{i}^{\text{I}}\left(t\right)=1$. The stochastic variables $s_{0}^{\text{E}}\left(t\right)$, $a_{0}^{\text{E}}\left(t\right)$, $x_{0}^{\text{E}}\left(t\right)$, and $u_{0}^{\text{E}}\left(t\right)$ for the excitatory subnetwork and $s_{0}^{\text{I}}\left(t\right)$, $a_{0}^{\text{I}}\left(t\right)$, $x_{0}^{\text{I}}\left(t\right)$, and $u_{0}^{\text{I}}\left(t\right)$ for the inhibitory subnetwork, and the macroscopic variables, $m_{0}^{\text{E}}\left(t\right)$, $A_{0}^{\text{E}}\left(t\right)$, $X_{0}^{\text{E}}\left(t\right)$, and $U_{0}^{\text{E}}\left(t\right)$ for the excitatory subnetwork and $m_{0}^{\text{I}}\left(t\right)$, $A_{0}^{\text{I}}\left(t\right)$, $X_{0}^{\text{I}}\left(t\right)$, and $U_{0}^{\text{I}}\left(t\right)$ for the inhibitory subnetwork are displayed as time courses in the thin solid, the thick solid, the thin dashed, and the thick dashed lines, respectively. **(C)** The power spectra of variables $s_{0}^{\text{I}}\left(t\right)$ and $m_{0}^{\text{I}}\left(t\right)$. The arrow indicates the representative frequency component with a high frequency. **(D)** The closed curves in the state space. **(E)** The slice, where section $a_{0}^{\text{I}}=1.7$ or $A_{0}^{\text{I}}=1.7$ with width ϵ = 0.001 was used. The ST0 appears in the slice because the emergent attractor is the MT1 (Komuro et al., 2016). Both the excitatory and the inhibitory subnetworks exhibit fast oscillations on the closed curve, whereas the amplitude of the oscillations on the excitatory subnetwork is small.

**Figure 10:**
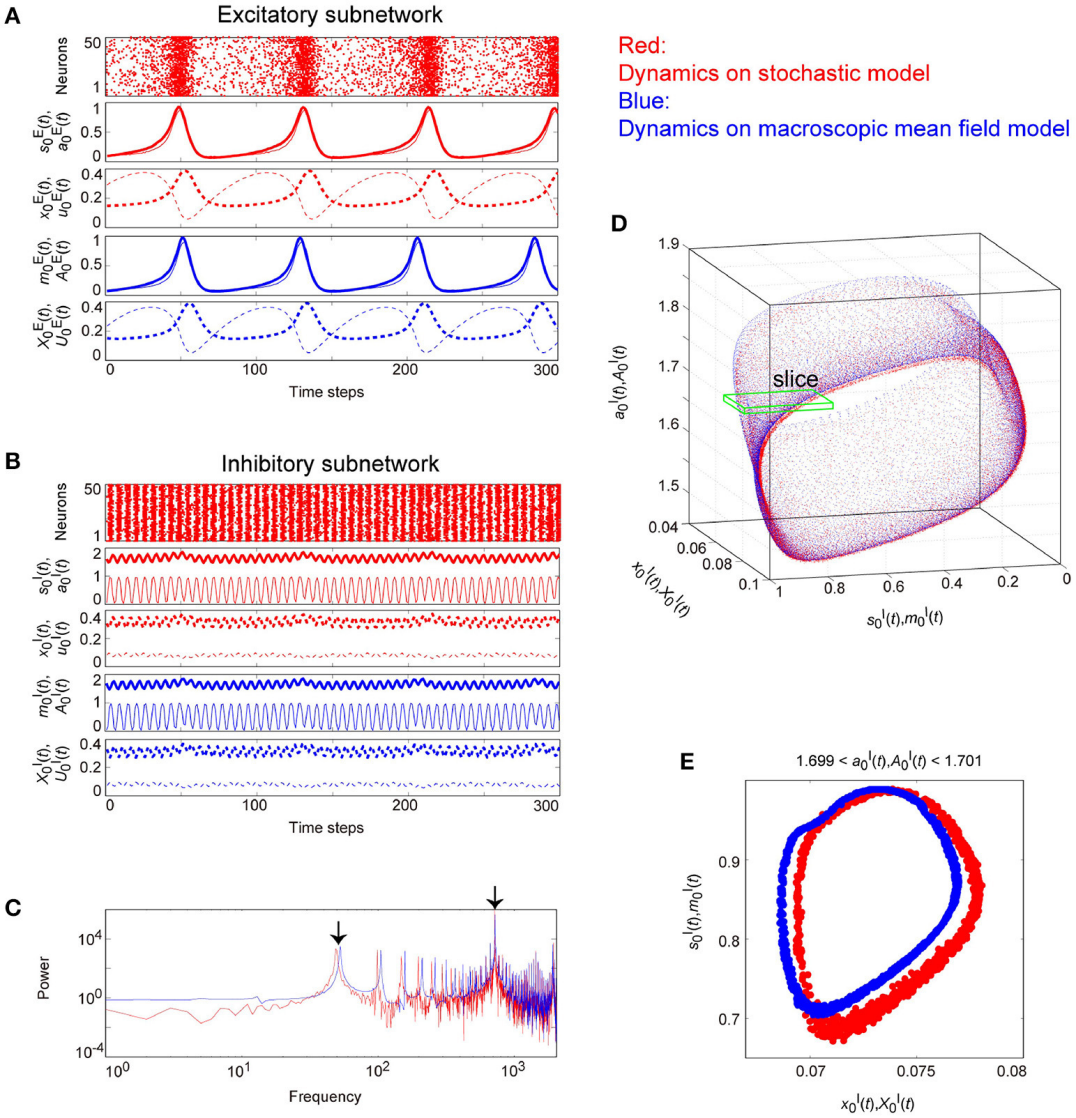
**The typical dynamics emergent from the OS2T state in the network with dynamic synapses**. The parameter set, $\left(J_{0}^{\text{EI}},J_{0}^{\text{IE}}\right)=\left(-0.05,2\right)$ for the bidirectional coupling between the excitatory and the inhibitory subnetworks, was used; other parameter sets are the same as those in Figure 9. The format of each panel is likewise the same as that in Figure 9. **(A,B)** The raster plots and the time courses of variables included in the stochastic and the macroscopic mean field models. **(C)** The power spectra of variables $s_{0}^{\text{I}}\left(t\right)$ and $m_{0}^{\text{I}}\left(t\right)$. The two arrows indicate the representative low and high frequency components. **(D)** The two-dimensional torus in the state space. **(E)** The slice, where section $a_{0}^{\text{I}}=1.7$ or $A_{0}^{\text{I}}=1.7$ with width ϵ = 0.001 was used. The ST1 appears in the slice because the emergent attractor is the MT2 (Komuro et al., 2016). The excitatory and the inhibitory subnetworks exhibit slow and fast oscillations on the torus, respectively, whereas, the amplitude of the fast oscillations, $s_{0}^{\text{I}}\left(t\right)$ and $m_{0}^{\text{I}}\left(t\right)$ on the inhibitory subnetwork is less modulated by the phase of the slow oscillations on the excitatory subnetwork.

**Figure 11:**
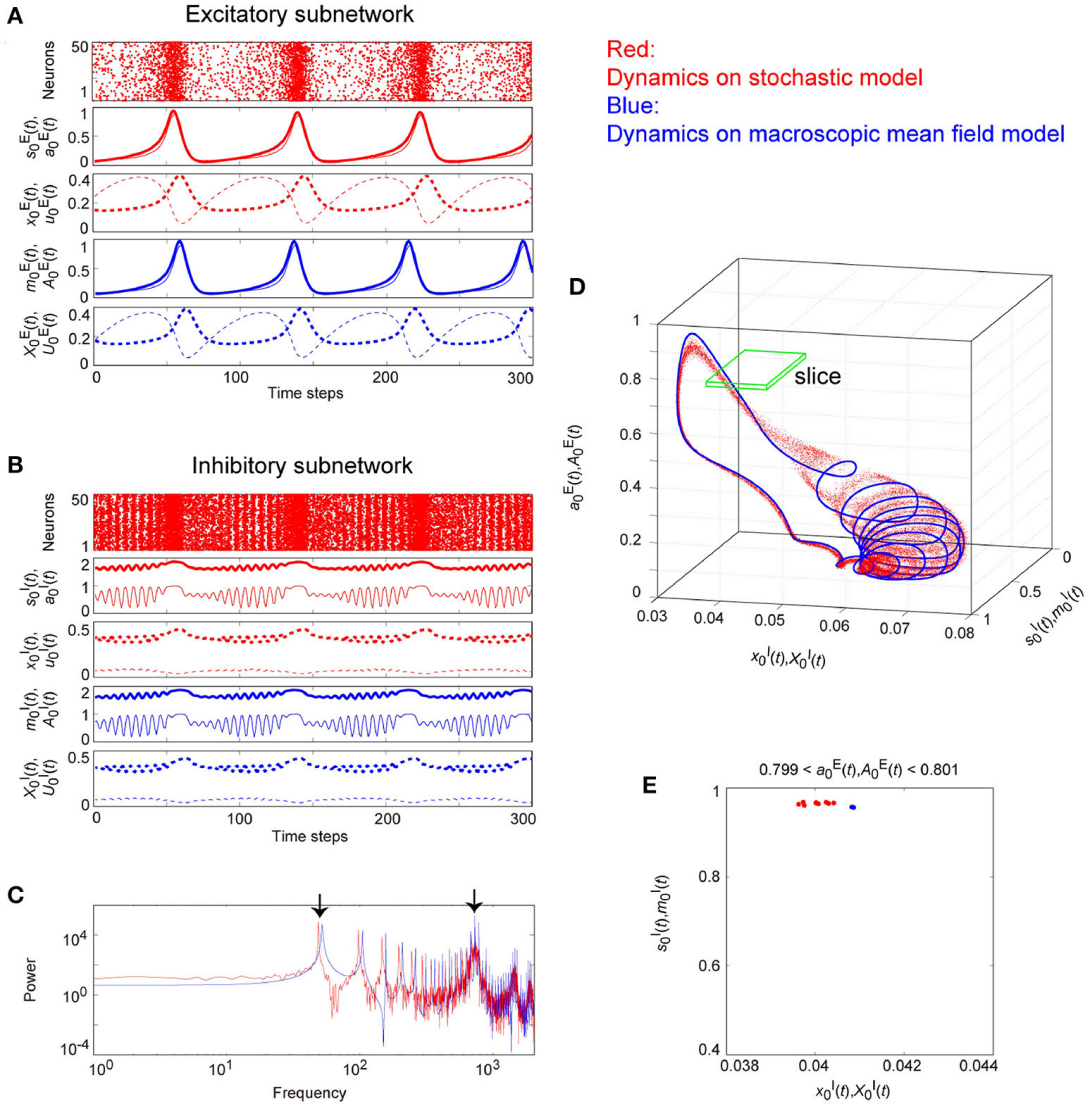
**The typical dynamics emergent from the OS2C state in the network with dynamic synapses**. The parameter set, $\left(J_{0}^{\text{EI}},J_{0}^{\text{IE}}\right)=\left(-0.05,5\right)$ for the bidirectional coupling between the excitatory and the inhibitory subnetworks, was used; other parameter sets are the same as those in Figure 9. The format of each panel is likewise the same as that in Figure 9. **(A,B)** The raster plots and the time courses of variables included in the stochastic and the macroscopic mean field models. **(C)** The power spectra of variables $s_{0}^{\text{I}}\left(t\right)$ and $m_{0}^{\text{I}}\left(t\right)$. The two arrows indicate the representative low and high frequency components. **(D)** The closed curve in the state space. **(E)** The slice, where section $a_{0}^{\text{E}}=0.8$ or $A_{0}^{\text{E}}=0.8$ with width ϵ = 0.001 was used. The ST0 appears in the slice because the emergent attractor is the MT1 (Komuro et al., 2016). The excitatory and the inhibitory subnetworks exhibit slow and fast oscillations on the closed curve, respectively, while, the amplitude of the fast oscillations, $s_{0}^{\text{I}}\left(t\right)$ and $m_{0}^{\text{I}}\left(t\right)$ on the inhibitory subnetwork is evidently modulated by the phase of the slow oscillations on the excitatory subnetwork.

**Figure 12:**
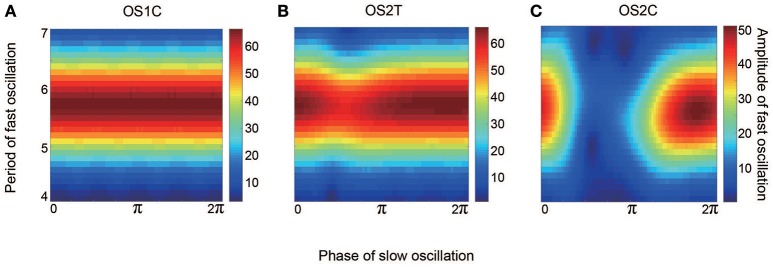
**Effect of the slow oscillation phase on the amplitude and on the period of fast oscillations, for the typical (A)** OS1C, **(B)** OS2T, and **(C)** OS2C states. The parameter sets for these states are the same as those in Figures 8–10, respectively. The amplitude of the fast oscillation in the OS1C state does not depend on the phase of the slow oscillation for any period, but this dependence appears in both the OS2T and the OS2C states around period 5.7. In particular, amplitude modulation arising from the OS2C state occurs more evidently compared to that in the OS2T state.

To help the readers to understand the bifurcation analysis results below, we summarize the Result section briefly here referring to Figures 3–**12**. First, we analyze the excitatory and inhibitory networks independently, so that a parameter space generating the oscillatory state becomes clear (Figures 3, **5**). The oscillation on the inhibitory network is faster than that on the excitatory network (Figure **6**), and the frequency of both oscillations changes depending on parameters *I*, τ_a_, and τ_R_/τ_F_ (Figures 3, **5**). Next, we analyze a network composed of the above two subnetworks, so that a parameter space generating two types of phase-amplitude CFCs becomes clear (**Figure 7**). The two CFC modes differ in the underlying attractors (**Figures 8**, **10**, **11**) and in the modulation properties (**Figure 12**), depending on parameters $J_{0}^{\text{EI}}$ and $J_{0}^{\text{IE}}$ (**Figure 7**), but this coupling phenomenon disappears if inhibitory input is large enough (**Figures 7**, **9**). While these analyses are applied to the macroscopic mean field model, the stochastic model also yields the consistent results (Figure 4).

The excitatory and the inhibitory networks, each of which reflects the property of depression synapses with τ_R_/τ_F_ = 11.7, exhibit distinctive oscillatory states, as shown in the (*J*_0_, *I*) phase diagram (Figure 3A). The oscillatory state on the excitatory network (OSE), which was already found in the previous model of Katori et al. (2012), changes into the steady state (the fixed point) via the Neimark-Sacker (NS) bifurcation (Kuznetsov, 2004), when *J*_0_ decreases/increases or *I* increases from the region of the OSE state. On the other hand, when *J*_0_ decreases and *I* increases from the region of the steady state, the oscillatory state on the inhibitory network (OSI) is likewise generated by the NS bifurcation; this OSI state has been newly observed here. It is clear from the bifurcation diagrams that these oscillatory states emerge via the NS bifurcation from the steady state (see Figures 3B,C). The bifurcation diagram, with respect to *J*_0_ on (*I*, τ_a_) = (−1, 2.5) (Figure 3B), shows the OSE state emergent from the steady state. The mean neural activity, *m*_0_, in the steady state, increases with an increase in *J*_0_, whereas the steady state destabilizes via the first NS bifurcation at *J*_0_ = 1.63 and then, the OSE state appears. Because of the second NS bifurcation at *J*_0_ = 3.48, the OSE state disappears, and accordingly, the steady state reappears. In contrast, the bifurcation diagram, with respect to *J*_0_ on (*I*, τ_a_) = (1, 2.5) (Figure 3C), shows the OSI state emergent from the steady state. The mean neural activity, *m*_0_, in the steady state decreases with a decrease in *J*_0_, whereas the steady state destabilizes via the NS bifurcation at *J*_0_ = −4.73 and accordingly, the OSI state appears. The OSE and the OSI states, generated from the steady state via the NS bifurcation, likewise appear on the network with facilitation synapses. The aforementioned two bifurcation diagrams, with respect to *J*_0_, show good agreement with those generated from the stochastic model (Figures 4A,B).

The model has been proposed as a discrete-time system, such that time is represented by an arbitrary unit to flexibly describe real data. To characterize oscillatory time-scale from this kind of time, first, we converted time courses generated from the model into the power spectrum, where 4096 time steps were used in calculation for a time course. Second, the frequency of the first typical peak in the spectrum was translated into the corresponding time steps; we call this time step value as “period”. Based on the period, we can say that the oscillation on the inhibitory network tends to be faster than that on the excitatory network (Figure 3A). Specifically, the period of the OSI state ranges from 4.99 to 6.00 time steps, whereas that of the OSE state ranges from 33.9 to 78.8 time steps.

Figures 3D,E show the bifurcation structures of the excitatory and the inhibitory networks, respectively, and represent the quasi-periodic oscillations on the invariant closed curve for both the OSE and the OSI states. The (τ_a_, *I*) phase diagram in Figure 3D shows the distribution of the OSE state, where the period of the OSE state ranges from 44.0 to 75.9 time steps and increases as τ_a_ and *I* decrease. In contrast, the (τ_a_, *I*) phase diagram in Figure 3E shows the distribution of the OSI state, where the period of the OSI state ranges from 3.94 to 8.00 time steps and tends to increase as τ_a_ and *I* increase.

The property of the dynamic synapses similarly affects the period on both the excitatory and the inhibitory networks (Figure 5). As the influence of short-term depression on the excitatory network increases (as τ_R_/τ_F_ increases), the period for the OSE state increases, and the network can oscillate with less inhibitory input (Figure 5A). In contrast, τ_R_/τ_F_ does not have much effect on the period for the OSI state (Figure 5B).

The typical OSE and OSI states represent the relatively slow and the fast oscillations, respectively, and are observed in both the stochastic and the macroscopic mean field models (Figure 6). The population averages of stochastic variables, $s_{0}\left(t\right)\left[=\left(1/N\right)\overset{N}{\underset{i}{\sum }}s_{i}\left(t\right)\right]$, $a_{0}\left(t\right)\left[=\left(1/N\right)\overset{N}{\underset{i}{\sum }}a_{i}\left(t\right)\right]$, $x_{0}\left(t\right)\left[=\left(1/N\right)\overset{N}{\underset{i}{\sum }}x_{i}\left(t\right)\right]$, and $u_{0}\left(t\right)\left[=\left(1/N\right)\overset{N}{\underset{i}{\sum }}u_{i}\left(t\right)\right]$, correspond to macroscopic variables (Figures 6A,D). Most excitatory (inhibitory) neurons fire together at each time step at a low (high) frequency, such that the macroscopic variables exhibit oscillations with large amplitude. The attractor, composed of the variables, is the closed curve for both the excitatory and the inhibitory networks (Figures 6C,F), but fundamental frequency components differ between these networks (Figures 6B,E). The dynamics of such macroscopic variables shows good agreement with that of the stochastic variables, in terms of: the time course (Figures 6A,D); the power spectrum (Figures 6B,E); and the trajectory in the state space (Figures 6C,F).

Figure 7 shows the dynamical structure of a network composed of the abovementioned excitatory and the inhibitory subnetworks, where the following parameter set, corresponding to the typical OSE and OSI states described above, has been used: $\left(J_{0}^{\text{EE}},I^{\text{E}},\tau_{\text{a}}^{\text{E}}\right)=\left(2,-1,2.5\right)$ for the excitatory subnetwork, $\left(J_{0}^{\text{II}},I^{\text{I}},\tau_{\text{a}}^{\text{I}}\right)=\left(-10,1,12.5\right)$ for the inhibitory subnetwork, and $\tau_{\text{R}}^{\xi }/\tau_{\text{F}}^{\xi }=11.7$ for the depression synapses. This network with $\left(J_{0}^{\text{EI}},J_{0}^{\text{IE}}\right)=\left(0,0\right)$ is a direct product system, composed of the excitatory and the inhibitory subnetworks, so that a part of the system is equivalent to the previous model of Katori et al. (2012). However, the network with $\left(J_{0}^{\text{EI}},J_{0}^{\text{IE}}\right)\neq \left(0,0\right)$ shows the following four types of distinctive neural dynamics: the steady state (SS); the oscillatory state with a single frequency component on a closed curve (OS1C); that with two frequency components on a two-dimensional torus (OS2T); and that with two frequency components on a closed curve (OS2C), where the OS2T and OS2C states did not appear in the previous model. It is clear from the number of zero-Lyapunov exponents whether the attractor underlying the network is a closed curve or a two-dimensional torus (see the $\left(J_{0}^{\text{EI}},J_{0}^{\text{IE}}\right)$ phase diagram in Figure 7A). The network dynamics involves only one zero-exponent for the emergence of the OS1C or the OS2C state, whereas the dynamics involves two zero-exponents for the emergence of the OS2T state. The dynamics is not affected by the property of the dynamic synapses characterized by parameter $\tau_{\text{R}}^{\xi }/\tau_{\text{F}}^{\xi }$, which merely changes the period of oscillations on subnetwork ξ.

Here we use the term MT*d* to investigate the bifurcation structure distinguishing the above four states clearly. Then, the MT1 arising from the model corresponds to the OS1C or the OS2C state, while the MT2 corresponds to the OS2T state. While the OS1C and the OS2C states are generated from the SS state via the NS bifurcation, these states change into the OS2T state via the two types of bifurcation, namely, the NS bifurcation of MT1 (MT1NS) and the saddle-node cycle bifurcation of MT1 (MT1SNC) (Kamiyama et al., 2014; Komuro et al., 2016). Because the MT1NS bifurcation here is subcritical, a saddle-node bifurcation of MT2 (MT2SN) intermediates between the OS1C and the OS2T states (Komuro et al., 2016), where there exists a specific hysteresis region between these states. We show below the qualitative difference between the states, before and after the bifurcation of the MT1 and the MT2, by observing the bifurcation diagrams generated from quasi-periodic points collected in the slice.

First, we consider the bifurcations intermediating between the OS1C and the OS2T states. The bifurcation diagram of trajectories in the slice, with respect to $J_{0}^{\text{EI}}$ on $J_{0}^{\text{IE}}=2$, shows a transition between the OS1C and the OS2T states, where section $A_{0}^{\text{I}}\left(t\right)=1.7$ and width ϵ = 0.001 have been used for the slice (Figure 7B). As $J_{0}^{\text{EI}}$ increases from $J_{0}^{\text{EI}}=-0.145$, the ST0, corresponding to the OS1C state, destabilizes and immediately an oscillation starts along with a jump to the ST1, corresponding to the OS2T state; this abrupt change is attributed to the subcritical bifurcation. The bifurcation property becomes clearer by a simultaneous observation of both trajectories in the slice, applied to a state just before the bifurcation, and that just after the bifurcation. This simultaneous analysis clarifies that the ST1 appears sufficiently far from the ST0 when the bifurcation occurs, where $J_{0}^{\text{EI}}=-0.142$ for the OS1C state, and $J_{0}^{\text{EI}}=-0.139$ for the OS2T state (Figure 7F). In contrast, as $J_{0}^{\text{EI}}$ decreases from $J_{0}^{\text{EI}}=-0.135$, the stable ST1 and the saddle ST1 collide, and the ST0 appears. By combining the aforementioned slice analysis with the Lyapunov exponent analysis, we can identify the bifurcation types more clearly (Figure 7D). The negative exponents with multiplicity of two approach zero simultaneously as $J_{0}^{\text{EI}}$ increases from $J_{0}^{\text{EI}}=-0.145$; this is a property of the NS bifurcation. However, only one exponent starts to decrease as $J_{0}^{\text{EI}}$ decreases from $J_{0}^{\text{EI}}=-0.135$; this is a property of the saddle-node bifurcation. Taken together, the bifurcation from the OS1C to OS2T states can be identified as the subcritical NS bifurcation of ST0 (ST0NS), while that from the OS2T to OS1C states as the saddle-node bifurcation of ST1 (ST1SN), within the slice (Komuro et al., 2016). Thus, these two bifurcations can be interpreted as the MT1NS and the MT2SN bifurcations, respectively, outside the slice (Komuro et al., 2016).

Next, we consider the bifurcation intermediating between the OS2C and the OS2T states. The bifurcation diagram of trajectories in the slice, with respect to $J_{0}^{\text{IE}}$ on $J_{0}^{\text{EI}}=-0.05$, shows a transition between the OS2C and the OS2T states, where section $A_{0}^{\text{E}}\left(t\right)=0.8$ and width ϵ = 0.001 have been used for the slice (Figure 7C). As $J_{0}^{\text{IE}}$ decreases from $J_{0}^{\text{IE}}=9$, the ST0, corresponding to the OS2C state, changes into the ST1 at a bifurcation point. It is clear that there does not exist a hysteresis region between the OS2C and the OS2T states, because the ST1 changes into the ST0 at the same bifurcation point as $J_{0}^{\text{IE}}$ increases from $J_{0}^{\text{IE}}=0$. By observing both a state just before the bifurcation and that just after the bifurcation simultaneously, we can find that the ST1 rightly covers the ST0, where $J_{0}^{\text{IE}}=3.12$ for the OS2C state and $J_{0}^{\text{IE}}=3$ for the OS2T state (Figure 7G). The bifurcation involving this feature has been recently identified as the saddle-node cycle bifurcation of ST0 (ST0SNC), within the slice (Figure 8A) (Komuro et al., 2016), or as the MT1SNC bifurcation, outside the slice (Figure 8B) (Komuro et al., 2016). Before the ST0SNC (MT1SNC) bifurcation occurs, there exists a pair of a stable ST0 (MT1) and a saddle ST0 (MT1) and therefore, an unstable set of the saddle ST0 (MT1) generates a one-dimensional (two-dimensional) torus. The ST0SNC (MT1SNC) bifurcation, where the stable ST0 (MT1) and the saddle ST0 (MT1) collide, stabilizes the unstable set and accordingly, an ST1 (MT2) appears. The mechanism of this MT1SNC bifurcation can be likewise verified by the Lyapunov exponent analysis (Figure 7E); only one negative exponent approaches zero as $J_{0}^{\text{IE}}$ decreases from $J_{0}^{\text{IE}}=9$.

Figures 9–**11** show typical dynamics emergent from the OS1C, the OS2T, and the OS2C states, respectively, where each is observed in both the stochastic and the macroscopic mean field models. The population averages of stochastic variables, $s_{0}^{\xi }\left(t\right)\left[=\left(1/N_{\xi }\right)\overset{N_{\xi }}{\underset{i}{\sum }}s_{i}^{\xi }\left(t\right)\right]$, $a_{0}^{\xi }\left(t\right)\left[=\left(1/N_{\xi }\right)\overset{N_{\xi }}{\underset{i}{\sum }}a_{i}^{\xi }\left(t\right)\right]$, $x_{0}^{\xi }\left(t\right)\left[=\left(1/N_{\xi }\right)\overset{N_{\xi }}{\underset{i}{\sum }}x_{i}^{\xi }\left(t\right)\right]$, and $u_{0}^{\xi }\left(t\right)\left[=\left(1/N_{\xi }\right)\overset{N_{\xi }}{\underset{i}{\sum }}u_{i}^{\xi }\left(t\right)\right]$, correspond to macroscopic variables. The dynamics of the macroscopic variables shows good agreement with that of the stochastic variables, in terms of: the time course (Figures 9A,B, 10A,B, 11A,B); the power spectrum (Figures 9C, 10C, 11C); the trajectory in the state space (Figures 9D, 10D, 11D); and its distribution in the slice (Figures 9E, 10E, 11E).

Figure 9 shows the appearance of the OS1C state in the network dynamics. Excitatory neurons in this state fire less coherently, such that the excitatory subnetwork does not show slow oscillations, but exhibits fast fluctuations (Figure 9A). In contrast, inhibitory neurons fire coherently at a high frequency, such that the inhibitory subnetwork shows the fast oscillation (Figure 9B). The fast fluctuation/oscillation can be generated with external inhibitory input and with a connection from the inhibitory to excitatory subnetworks. These two types of inhibition are needed for the generation of the OS1C state. The fast oscillation in this state exhibits a single representative peak (Figure 9C), indicating that the amplitude of the oscillation is not modulated by the phase of the excitatory oscillation (Figures 9B, 12A). The dynamics emergent from both the excitatory and the inhibitory neurons exhibits a closed curve (Figure 9D). Accordingly, its slice includes less points, forming an ST0 (Figure 9E).

Figure 10 shows the appearance of the OS2T state in the network dynamics. Both excitatory and inhibitory neurons in this state fire coherently, such that all variables can show oscillations, whereas, the frequency differs between the excitatory and the inhibitory subnetworks. Excitatory oscillations are relatively slower than inhibitory ones (Figures 10A,B). The inhibitory fast oscillation exhibits two representative peaks (Figure 10C), indicating that the oscillation amplitude is slightly modulated by the phase of the excitatory slow oscillation (Figures 10B, 12B). This modulated oscillation is a phenomenon of phase-amplitude CFC and appears continuously (Figure 10B). Therefore, we call this as continuous CFC following Hyafil et al. (2015). The dynamics composed of both the slow and the fast oscillations exhibits a two-dimensional torus (Figure 10D). Accordingly, its slice includes a closed curve, forming an ST1 (Figure 10E).

Figure 11 shows the appearance of the OS2C state in the network dynamics. The firing properties of excitatory and inhibitory neurons and their macroscopic oscillations in this state are similar to those in the OS2T state (Figures 11A–C), where the slow and the fast oscillations appear in the excitatory and the inhibitory subnetworks, respectively. However, the amplitude of the fast oscillation is more evidently modulated by the phase of the slow oscillation, compared to the OS2T state (Figures 11B, 12C). This modulated oscillation is likewise a phenomenon of phase-amplitude CFC but appears intermittently (Figure 11B). Therefore, we call this as intermittent CFC following Hyafil et al. (2015). The dynamics composed of both the slow and the fast oscillations exhibits a complicated closed curve (Figure 11D), whereas, its slice includes a few points, forming an ST0 (Figure 11E).

## 4. Discussion

In this study, we analyzed a stochastic network model composed of excitatory and inhibitory neurons with dynamic synapses, and converted the model into the corresponding macroscopic mean field model. The bifurcation analysis of the mean field model revealed the overall dynamical properties of the network. The excitatory and the inhibitory subnetworks represent slow and fast oscillations, respectively. The interaction between these two subnetworks generates diverse oscillatory states with two major frequency components. This oscillatory phenomenon corresponds to phase-amplitude cross-frequency coupling (CFC). The dependence of the oscillatory states on coupling strengths, mediating between the subnetworks, has been clarified by the bifurcation analysis. Furthermore, it has been found that the oscillatory states of the CFC can be classified into two subtypes, namely, an oscillatory state with two frequency components on a two-dimensional torus (OS2T), which can generate the continuous CFC, and an oscillatory state with two frequency components on a closed curve (OS2C), which can generate the intermittent CFC.

The present model is an extension of an excitatory neural network model with dynamic synapses (Katori et al., 2012). The previous model, which corresponds to the excitatory subnetwork in this study, was modified in terms of the following three aspects: First, we analyzed the dependence of the network dynamics on the coupling strength, $J_{0}^{\xi \xi }$, and on the external input *I*^ξ^; these parameters were fixed in the previous study (Katori et al., 2012). The analysis revealed that these two parameters can be crucial for the generation of a variety of oscillatory states. The second point is the introduction of an additional variable, $a_{i}^{\xi }\left(t\right)$, corresponding to the synaptic activity, and a parameter $\tau_{\text{a}}^{\xi }$, corresponding to the time constant of the decay process for $a_{i}^{\xi }\left(t\right)$. The third point is a combination of the excitatory network with the inhibitory one, where parameters $J_{0}^{\text{EI}}$ and $J_{0}^{\text{IE}}$ were introduced.

Depending on the synaptic properties, the network dynamics changes. Decay time constants, $\tau_{\text{a}}^{\text{E}}$ and $\tau_{\text{a}}^{\text{I}}$ of the synaptic activity, may determine the frequency of slow oscillations in the excitatory network and that of fast oscillations in the inhibitory network, respectively (Figures 3D,E). The frequency can be likewise changed by $\tau_{\text{R}}^{\xi }/\tau_{\text{F}}^{\xi }$, the property of short-term plasticity (Figures 5A,B). A variation of frequency bands in neural activity, such as the delta, theta, alpha, beta, and gamma bands, is often observed in the brain, and it has been suggested that this variation correlates with the brain functions (Buzsáki and Draguhn, 2004). The brain functions may be attributed to the above synaptic parameters. Indeed, aminomethylphosphonic acid (AMPA) synapses have a relatively short time constant, whereas, N-methyl-D-aspartate (NMDA) synapses have a longer time constant. This synaptic difference should affect the generation of neural oscillations and brain functions.

We have found that the generation mechanism of the OSI state on the inhibitory subnetwork is qualitatively consistent with the physiological experiments (Fisahn et al., 1998; Mann et al., 2005). Inhibitory interneurons in the rat hippocampal CA3 region show a fast oscillation, which is referred to as the gamma oscillation. This oscillation is blocked by the AMPA or the gamma-aminobutyric acid (GABA) type-A receptor antagonist. AMPA-type synapses send excitatory input to the interneurons, while GABA type-A synapses send recurrent inhibitory connections. These antagonists can be considered as the realization of input *I*^I^ and the absolute value of coupling strength $J_{0}^{\text{II}}$, respectively (Figure 3A). Taken together, the OSI state generated from the present model can be related to the gamma oscillation in inhibitory interneuron networks.

The network composed of both excitatory and inhibitory neurons shows phase-amplitude CFC, in which the amplitude of the fast oscillation is modulated by the phase of the slow oscillation (Figures 10B, 11B, 12B,C). The property of this oscillatory phenomenon is similar to that of the experimentally known CFC between the theta and the gamma oscillations observed in the entorhinal cortex of the hippocampus (Chrobak and Buzsáki, 1998).

Various oscillatory phenomena, including CFC arising from our model, may contribute to information coding in the brain. The presence of distinctive oscillatory states in the model implies that a variety of information coding schemes can exist in brain networks. It has been shown that the oscillatory states with two major frequency components can be classified into two subtypes, OS2T and OS2C states (Figures 10, 11). In the OS2T state, peaks of the fast oscillation are broadly distributed in the phase of the slow oscillation (Figure 10B). In contrast, in the OS2C state, the phase of the fast oscillation is partially locked by the slow oscillation; that is, peaks of the fast oscillation appear in specific phases of the slow oscillation (Figure 11B). The neural activity phase can be utilized to encode certain information. Indeed, it has been suggested that the physiologically observed CFC provides a basis for effective communications among neurons (Chrobak and Buzsáki, 1998).

The OS2C state may contribute to multi-item representation (Hyafil et al., 2015), because this state can generate the intermittent CFC in the inhibitory subnetwork. One cycle of the fast oscillation of the CFC would correspond to one item, associated with the working memory, the spatial memory, or the visual attention. Owing to intermittency of this oscillation, external multi-items could be effectively encoded in the brain; i.e., the slow oscillation, generated from the excitatory subnetwork, would play a role in optimizing storage capacity for the items. The typical fast oscillation, $m_{0}^{\text{I}}\left(t\right)$, generating the CFC in the OS2C state depicted in Figure 11B, shows that approximately 11 items are possibly encoded. The number of items to be stored may increase or decrease depending on parameters, *I*^E^ and *I*^I^, related to, e.g., visual input, because these parameters affect the frequency of the slow and the fast oscillations, respectively (Figures 3D,E). In contrast, the OS2T state would not be suitable for multi-item representation because this state generates continuous CFC. Moreover, the encoding scheme for the multi-items can be likewise observed on the closed curve underlying the fast oscillation $m_{0}^{\text{I}}\left(t\right)$ in the OS2C state (Figure 11D); that is, the number of items to be stored would be limited in order for the brain to avoid producing wasted storage capacity. In contrast, the attractor underlying the OS2T state is a two-dimensional torus (Figure 10D); that is, more items would be encoded in the torus than the closed curve. However, the torus may not be efficient where only a few items are stored, because the torus consists of a dense orbit and may produce wasted storage capacity.

Our main finding is that the MT1SNC bifurcation may underlie a switching phenomenon between the continuous and the intermittent CFCs; this result supports the study of Fontolan et al. (2013). Fontolan et al. have reported that these two CFCs are switched via the saddle-node on invariant circle (SNIC) bifurcation, on a simplified Pyramidal Interneuron Network Gamma (PING) model (Fontolan et al., 2013). The SNIC bifurcation mediates between a limit cycle and a two-dimensional torus in flow, whereas the MT1SNC bifurcation mediates between a closed curve and a two-dimensional torus in map; here both bifurcations occur via a saddle-node cycle. If we assume that the MT1 arising from the proposed discrete-time model is the limit cycle in the corresponding continuous-time model, these two bifurcations will become consistent. Thus, the present study implies that phase-amplitude CFC in the brain can be interpreted in a discrete-time model.

Overall, the bifurcation analysis revealed that oscillatory dynamics, arising from the proposed model, qualitatively changes depending on parameters, which would be one origin of characterizing cell assemblies. Although the present study focused only on one cell assembly receiving inhibitory and external input, in fact, there exist many assemblies (Yoshimura et al., 2005), which could differ in their roles for neural information processing. Each of assemblies, in layer 2/3 of the cortex, selectively receives its recurrent connections and excitatory input from layer 4, possibly based on environmental change, while input from layer 5 might modulate activity between assemblies (Yoshimura et al., 2005). Such selectively interconnected neurons would play a crucial role for utilizing phase-amplitude CFC in the brain.

The mechanisms and functions of oscillatory phenomena must be further explored in the future. The oscillatory phenomena observed in the proposed model, a binary-state discrete-time neuron model, should be evaluated with a more realistic network model that reflects the detailed properties of the cerebral cortex.

## Author contributions

YK and TS modeled neural networks with dynamic synapses. MK checked the details of the bifurcation analysis. KA supervised the overall research. TS analyzed the details of the model and did all other things.

### Conflict of interest statement

The authors declare that the research was conducted in the absence of any commercial or financial relationships that could be construed as a potential conflict of interest.
